# Dibenzodioxin-Based Polymers of Intrinsic Microporosity with Enhanced Transport Properties for Lithium Ions in Aqueous Media

**DOI:** 10.1021/acs.macromol.4c01243

**Published:** 2024-09-27

**Authors:** Juan Carlos Martínez-López, Marta Santos Rodríguez, Víctor Oliver Cuenca, Giu Silva Testa, Ernst van Eck, Evan Wenbo Zhao, Ángel E. Lozano, Cristina Álvarez, Javier Carretero-González

**Affiliations:** † Institute of Polymer Science and Technology, ICTP, CSIC, C/Juan de la Cierva, 3, Madrid 28006, Spain; ‡ Magnetic Resonance Research Center, Institute for Molecules and Materials, Radboud University, Nijmegen, AJ 6525, The Netherlands

## Abstract

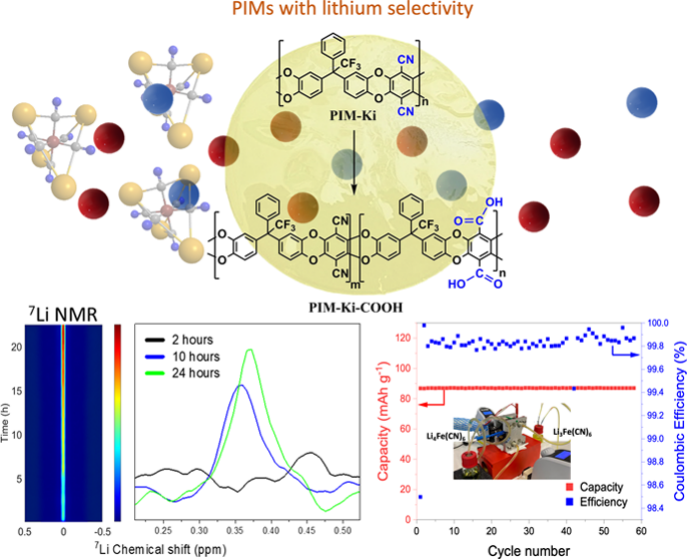

Boosting the transport and selectivity properties of membranes based on polymers of intrinsic microporosity (PIMs) toward one specific working analyte of interest is challenging. In this work, a novel family of PIM membranes, prepared by casting and exhibiting optima mechanical properties and high thermal stability, was synthesized from 4,4′-(2,2,2-trifluoro-1-phenylethane-1,1-diyl) bis(benzene-1,2-diol) and two tetrafluoro-nitrile derivatives. Gas permeability measurements evidenced a CO_2_/CH_4_ selectivity up to 170% relative to the reference polymer, PIM-1, in agreement with their calculated fractional free volume and the analysis of the textural properties by N_2_ and CO_2_ gas adsorption. Besides, the chemical modification by acid hydrolysis of the PIM membranes favored the permeability for lithium ions (LiCl 2M, 6 × 10^–9^ cm^2^·s^–1^) compared to other alkali metal analogs such as sodium (NaCl 2M, 7.38 × 10^–10^ cm^2^·s^–1^) and potassium (KCl 2M, 1.05 × 10^–9^ cm^2^·s^–1^). Moreover, the complete mitigation of the crossover of redox species with higher molecular sizes than the ions from alkali metal salts was confirmed by using *in-line* benchtop NMR methods. Additionally, the modified PIM membranes were measured in a symmetric electrochemical flow cell using an aqueous electrolyte by combining lithium ferro/ferricyanide redox compounds and lithium chloride. The electrochemical tests showed low polarization, high-rate capability, and capacity retention values of 99% when cycled at 10 mA·cm^–2^ for over 50 cycles. Based on these results, these polymers could be used as highly selective and conducting membranes in electrodialysis for lithium separation and lithium-based redox flow batteries and as a protective layer in high-energy density lithium metal batteries.

## Introduction

Polymers of intrinsic microporosity (PIMs) are an attractive class of *ladder* polymers with a combination of properties—such as good solubility in common solvents (chloroform and tetrahydrofuran), which facilitates film processing, high free volume, and excellent capacity to act as efficient molecular sieves—that have attracted significant attention in the field of materials science and engineering.^1−3^ The high porosity of PIMs arises from the inefficient packing of the chains due to their highly rigid and contorted molecular structure.^4,5^

The implementation of PIMs in specific applications is highly dependent on the design and synthesis of monomers to obtain polymers with the required porosity and functionalization. For example, PIMs have a pore size distribution that is selective to a specific analyte. Special efforts have been made in the synthesis and chemical postmodification of dibenzodioxin-based PIMs. Particularly, the use of 5,5′,6,6′-tetrahydroxy-3,3,3′,3′-tetramethylspirobisindane monomer (TTSBI) has allowed the creation of a plethora of dibenzodioxin-based PIMs with high free volume consisting of interconnected pores and specific interactions.^6^ These dibenzodioxin-based PIMs have been traditionally linked to the study of gas separation properties^4,7−9^ and the capture and purification of hydrogen,^10,11^ carbon dioxide, and other small molecules.^12,13^ Alternatively, PIMs can also be synthesized through other step-growth polymerization methods,^10^ such as the ring-opening metathesis polymerization (ROMP) or the Sonogashira coupling reaction.^10,12,14,15^

In the last years, an increasing number of studies evaluating the properties of PIMs for separating liquids^16^ and for application in energy storage systems^17−20^ have been published. Some of the relevant research includes the preparation of highly selective ion-conducting dibenzodioxin-based derivatives prepared from PIM-1 through the modification of the cyano groups to ionizable carboxylate and amidoxime moieties.^17,18^ The membranes exhibited optimum performance as a size-sieving molecular separator in aqueous redox flow batteries (RFBs).

Another groundbreaking research was recently found by recognizing that the functional groups of spirobisindane and dihydroethanoanthracene monomers can be modified via multicomponent Mannich reactions.^21^ The polymerization of these substrates with tetrafluoroterephthalonitrile rendered PIM membranes introducing a lithium-coordinating functionality inside the free-volume elements (i.e., ion-solvation cages) that exhibited high ionic conductivity and cation transfer number. One member of this PIM family when implemented as an anode-stabilizing interlayer in lithium–metal batteries suppressed the formation of dendrites.^21^ Further strategies for the creation of advanced PIM materials for energy storage applications have been based on the preparation of solid polymer electrolytes by blending poly(ethylene oxide) (PEO) and PIM-1, evidencing an increase of the mechanical properties, stability against metallic lithium, and high ionic conductivity when compared to regular PEO electrolytes. Membranes made of these composite materials have been implemented as safe solid electrolytes improving the electrochemical performance of lithium–sulfur batteries.^22^

Here we report the development of a novel family of PIMs (hereinafter referred to simply as PIM-Kis), which have been synthesized by aromatic nucleophilic substitution (S_N_Ar) polycondensation reactions.^2^ For that, the 4,4′-(2,2,2-trifluoro-1-phenylethane-1,1-diyl)-bis(benzene-1,2-diol) monomer (TPBB) has been used as an alternative monomer to the classical TTSBI monomer. Although the TPBB does not exhibit the typical molecular structure that leads to ladder-type polymers, we believe that the TPBB structure is rigid enough and that it would be capable of creating polymers with a PIM-type behavior. Thus, two homopolymers were obtained by combining the TPBB monomer^23^ and two tetrafluoro-nitrile derivatives to form dibenzodioxin-linked polymers. We also report the synthesis of a new copolymer derived from the TTSBI and the TPBB monomers using tetrafluoroterephthalonitrile (TFTPN) as the electrophilic monomer. As the interest in developing energy storage systems is increasing, we have focused efforts on the study of PIM-Kis as ion selective membranes in aqueous media. A postsynthetic acid hydrolysis reaction from TTSBI-based PIMs has been made to improve the polymer hydrophilicity and make it usable as a selective membrane with enhanced lithium-ion transport in aqueous media compared to other alkali metal cation analogs. Besides, we have studied by *in-line* NMR the lithium ion diffusion across membranes, evidencing the absence of crossover of redox-active molecules of a higher molecular size. Finally, the PIM membranes have been integrated in a symmetric flow cell by using lithium ferri/ferrocyanide salts, Li_3_Fe(CN)_6_)/Li_4_Fe(CN)_6_), as an aqueous redox electrolyte, where lithium is the working ion, to study their electrochemical reversibility and rate capability along with a capacity retention for a current density range of 10–40 mA·cm^–2^.

All these insights will aid the design of future PIM-based membranes with better conductivity and selectivity toward specific analytes to be applied in diverse fields, including but not limited to gas and liquid separation and purification processes, and in energy applications^17^ as redox flow systems.^24,25^

## Experimental Section

### Materials

Anhydrous potassium carbonate (K_2_CO_3_, Sigma-Aldrich) was heated at 160 °C for 3 days before use. 5,5′,6,6′-Tetrahydroxy-3,3,3′,3′-tetramethylspirobisin-dane (TTSBI, Sigma-Aldrich) was purified by crystallization in tetrahydrofuran (THF). Anhydrous toluene, anhydrous dimethylacetamide (DMAc), tetrafluoroterephthalonitrile (TFTPN), 2,3,5,6-tetrafluoroisonicotinonitrile (TFPyCN), trifluoromethanesulfonic acid (TFMS), 98% sulfuric acid (H_2_SO_4_) and glacial acetic acid were used as received (Sigma-Aldrich). Pyrocatechol and 2,2,2-trifluoroacetophenone (TFAP) were also used as received (Sigma-Aldrich) to synthesize the 4,4′-(2,2,2-trifluoro-1-phenylethane-1,1-diyl)-bis(benzene-1,2-diol) monomer (TPBB) through a modified reported method (additional details about the synthesis are found in Supporting Information Section 1.1).^23^ Lithium ferro/ferricyanide were obtained from ion exchange process from potassium ferro/ferricyanide salts (Sigma-Aldrich) using a Dowex 50 resin (more details in Supporting Information, Section 3.2, Figure S23).^26^ Hydrochloric acid (HCl), lithium hydroxide (LiOH), lithium chloride (LiCl), potassium chloride (KCl), and sodium chloride (NaCl) salts were used as received (Sigma-Aldrich).

### Synthesis of Polymers

#### Synthesis of PIM-1, PIM-Ki, and PIM-KiPY

The synthesis of these homopolymers followed a variation of the method based on a previous one reported by McKeown.^1^ The general procedure was as follows: TTSBI or TPBB (5 mmol), TFTPN or TFPyCN (5 mmol), and anhydrous DMAc (7.2 mL) were added to a 50 mL three-neck round-bottom flask equipped with a magnetic stirrer, a Dean–Stark apparatus, and a nitrogen inlet. The mixture was stirred for 10 min until the reactants were dissolved. After that, the solution was heated up to 60 °C by using a preheated silicon bath, and then the temperature was increased until 90 °C. Then, anhydrous K_2_CO_3_ (15.12 mmol) was added, and the formation of yellow bubbles was observed. The solution was immediately heated to 130 °C, and anhydrous toluene (5.9 mL) was added. At this point, the solution viscosity increased a lot, and small volumes of anhydrous toluene (5 mL each) were added to the solution during the reaction to enhance the stirring and to help solubilize as much nonreacted K_2_CO_3_ as possible and to eliminate the water produced during the polycondensation reaction. After 3 h, the temperature was stopped, and before reaching room temperature, the reaction mixture was poured into a methanol bath under magnetic stirring. The polymer precipitated as small, yellow fibers. The solid was vacuum filtered using a Büchner funnel and then washed with distilled water to remove the remaining salt. Then it was dissolved in chloroform (CHCl_3_), precipitated again from methanol, and dried at 100 °C overnight.

##### **PIM-1** (TTSBI-TFTPN)

Yield: 76%; Mw: 103,000 g·mol^–1^ and polydispersity index (PDI): 2.09; ^1^H NMR (400 MHz, CDCl_3_): δ (ppm) 6.74, 6.34, 2.25, 2.08, 1.26. ^13^C NMR (101 MHz, CDCl_3_): δ (ppm) 150.12, 147.37, 139.91, 139.65, 112.75, 110.98, 109.84, 94.54, 77.65, 59.26, 57.57, 44.05, 44.02, 31.80, 30.35. (C_33_H_32_N_2_O_4_) Theoretical: C, 75.64; H, 4.38; N, 6.08. Experimental: C, 72.78; H: 4.60; N, 5.83. (NMR spectra in Supporting Information, Section 1.2.1).

##### **PIM-**K**i** (TPBB-TFTPN)

Yield: 87%; Mw: 102,000 g·mol^–1^ and PDI: 1.98; ^1^H NMR (400 MHz, CDCl_3_): δ (ppm) 7.38, 7.37, 7.26, 7.12, 7.11, 7.00, 6.98, 6.96, 6.87, 6.85, 6.75, 1.46, 0.08. ^13^C NMR (101 MHz, CDCl_3_): δ (ppm) 139.50, 139.34, 139.30, 139.25, 138.23, 137.61, 129.56, 128.90, 127.39, 125.91, 118.88, 116.92, 109.10, 94.85, 94.73, 94.60, 77.48, 77.36, 77.16, 76.84, 64.40, 64.16, 51.02. (C_32_H_23_F_3_N_2_O_4_) Theoretical: C, 67.75; H, 2.23; N, 5.64. Experimental: C, 66.08; H: 2.57; N, 5.62. (NMR spectra in Supporting Information, Section 1.2.2).

##### **PIM-KiPY** (TPBB-TFPyCN)

Yield: 79%; Mw: 83,000 g·mol^–1^ and PDI: 1.97; ^1^H NMR (400 MHz, CDCl_3_): δ (ppm) 7.37, 7.27, 7.13, 7.11, 6.95, 6.92, 6.85, 6.82, 6.79, 6.75, 6.69. ^13^C NMR (101 MHz, CDCl_3_): δ (ppm) 142.07, 142.00, 140.84, 140.81, 140.77, 140.74, 139.74, 138.76, 137.63, 137.52, 135.32, 131.94, 129.91, 129.11, 127.45, 127.31, 126.25, 123.40, 119.52, 118.93, 117.43, 116.82, 109.07, 99.84, 99.77, 77.80, 77.48, 77.16, 64.64, 64.39, 64.16, 51.33, 1.47. (C_30_H_23_F_3_N_2_O_4_) Theoretical: C, 66.11; H, 2.35; N, 5.93. Experimental: C, 65.10; H: 2.66; N, 5.99. (NMR spectra in Supporting Information, Section 1.2.3).

#### Synthesis of the PIM1-PIMKi-CO-11 Copolymer

This copolymer was synthesized by a stoichiometric reaction of TTSBI, TPBB, and TFTPN (molar ratio 1:1:2) following the above procedure.

##### **PIM1-PIMKi-CO-11** (TTSBI-TPBB-TFNP)

Yield: 83%; Mw: 90,000 g·mol^–1^ and PDI 2.95; ^1^H NMR (400 MHz, CDCl_3_): δ (ppm) 7.38, 7.25, 7.11, 7.00, 6.98, 6.86, 6.81, 6.75, 6.47, 6.42, 2.33, 2.32, 2.18, 2.15, 1.61, 1.37, 1.31, 1.00. ^13^C NMR (101 MHz, CDCl_3_): δ (ppm) 149.99, 149.86, 147.20, 147.05, 139.76, 139.59, 139.48, 139.31, 138.83, 138.24, 137.60, 137.46, 129.54, 128.88, 127.37, 125.88, 123.03, 118.85, 116.90, 112.48, 110.71, 109.55, 109.33, 109.12, 94.83, 94.71, 94.57, 77.48, 77.16, 76.84, 64.37, 64.13, 58.92, 57.28, 51.04, 43.76, 31.49, 31.09, 30.04. (C_61_H_43_F_3_N_4_O_8_) Theoretical: C, 71.55; H, 3.27; N, 5.86. Experimental: C: 70.50; H: 3.68; N: 5.96. (NMR spectra in Supporting Information, Section 1.2.4).

#### Synthesis of PIM-Ki-COOH

A similar acid hydrolysis method to that reported by Mizrahi Rodriguez et al. was used to convert the cyano (CN) groups of PIM-Ki to carboxylic (COOH) groups.^27^ The procedure was as follows: PIM-Ki bright yellow powder (3 g), deionized water (100 mL), glacial acetic acid (40 mL), and concentrated sulfuric acid (100 mL) were charged into a 500 mL round-bottom flask equipped with a condenser and a magnetic stirrer. The mixture was refluxed at 150 °C, and the reaction time was varied from 50 to 150 h to obtain polymers with different hydrolysis degrees. Afterward, the mixture was allowed to cool to room temperature and poured into 500 mL of deionized water to neutralize the acid medium. The yellowish-brown powder was then vacuum filtered using a Büchner funnel, charged into a 500 mL round-bottom flask with 250 mL of deionized water and 10 drops of concentrated H_2_SO_4_, and left to reflux overnight to remove the residual reagents. Finally, the mixture was filtered under vacuum and dried at 130 °C overnight. A yellowish-brown powder was obtained.

##### PIM-Ki-COOH

^1^H NMR (400 MHz, DMSO-*d*_6_) δ (ppm) 13.89, 8.02, 7.75, 7.40, 7.04, 6.67, 6.65, 6.52, 6.40. ^13^C NMR (101 MHz, DMSO-*d*_6_) δ (ppm) 162.58, 140.36, 138.72, 135.95, 133.96, 129.67, 129.40, 126.61, 117.34, 114.11, 89.84, 63.83 (NMR characterization can be found in Supporting Information, Section 1.2.5). (C_60_H_36_F_6_N_2_O_12_) Theoretical: C, 67.50; H, 2.23; N, 0.2. Experimental: C, 58.98; H: 3.16; N, 0.51. Conversion ratio (reaction time): 65,2% (50 h) and 100% (150 h).

### Film Casting

The nonmodified or modified polymer (500–600 mg) was dissolved in 10–20 mL of THF overnight and then filtered through a glass microfiber (GMF) 3,1 mm filter. Afterward, it was poured onto a glass ring (11 cm diameter), which was placed on leveled glass to obtain a homogeneous thickness film. The solvent was allowed to evaporate at room temperature overnight. Subsequently, the film was peeled off the glass by immersing it in a water/ethanol bath, if needed. Films with 40–70 μm thicknesses were obtained, which were dried at 120 °C overnight (Figure S14, Supporting Information). The absence of solvent in the film was confirmed by TGA analysis. (Solubility tests and pictures of the films (Figure S14) are included in Supporting Information Section 1.3.)

### Characterization Methods

NMR spectra of monomer and polymers were recorded on a Bruker spectrometer at a resonance frequency of 400 MHz for ^1^H and 101 MHz for ^13^C NMR at room temperature. The samples (10–20 mg) were dissolved in 700 μL of deuterated chloroform (CDCl_3_) or deuterated dimethyl sulfoxide (DMSO-*d*_6_). The CDCl_3_ signal (^1^H 7.25 ppm, ^13^C 77.2 ppm) and DMSO-*d*_6_ signal (^1^H 2.5 ppm, ^13^C 40.6 ppm) were used as the internal reference.

Attenuated total reflectance-Fourier transform infrared (ATR-FTIR) spectra of polymers were obtained in a PerkinElmer RX-1 FTIR spectrometer equipped with an ATR accessory. The wavenumber window was 400 to 4000 cm^–1^, and an average spectrum of polymer was obtained from 64 scans·s^–1^.

Elemental analysis of polymers was carried out using an Elemental LECO CHNS-932 instrument with infrared (C, H, S) and thermic (N) detectors and a 0.001–100% and 0.01–100% detection range for H and S, respectively. Silver (3.3 mm diameter) and 4 mm high capsules were used. The amount of sample used was 1 mg.

Inductively coupled plasma mass spectrometry (ICP-MS, Agilent 7900) was used for the quantification of lithium (Li) and potassium (K) in the compounds after the ion exchange process. The samples were weighted with a Mettler Toledo UMX2 automated-s ultramicrobalance, and they were digested in a high-pressure microwave digestion system (Ethos Easy, Milestone). A scandium (^45^Sc) internal standard solution at 20 mg·g^–1^ from ISC Science was used. Certified individual standards of 10,000 and 1000 μg·mL^–1^ for K and Li, respectively, and high-purity standards were also used for the analysis. (The results can be found in Supporting Information, Section 3.2.1.)

Molecular weight and molecular weight distributions of PIMs were obtained by size exclusion chromatography (SEC) using a system coupled with a Waters 2414 refractive index detector. The measurements were performed on three (300 × 7.8 mm ID) Waters Styragel columns packed with 50, 100, and 10000 Å, 5 μm particle diameter. The injector and column compartments were maintained at 35 °C. Each sample was injected at a volume of 100 μL and run in THF with 0.1% LiBr at a flow rate of 1 mL·min^–1^ using a Waters 1515 Isocratic HPLC pump. The sample was prepared at a concentration of 4 mg·mL^–1^, and it was filtered through a 0.20 μm disposable Teflon filter (PTEF filter 0.2 μm, 17 mm, Symta) before injection. The calibration of the SEC system was performed using polystyrene standards with molecular weights ranging from 580 to 402,100 Da. Carboxylated PIMs were not measured because the presence of acid groups increases the retention time within the columns; thus, the molecular weights of these polymers were unreliable.

Thermogravimetric analysis (TGA) of the samples was carried out using a TA-Q500 thermobalance under a nitrogen atmosphere (60 mL·min^–1^). Dynamic TGA measurements were performed using the high-resolution TGA mode (Hi-RES TGA) with a heating rate of 20 °C·min^–1^ and sensitivity and resolution parameters of 1 and 4, respectively, covering a temperature range of 30 to 850 °C.

Wide-angle X-ray diffraction (WAXD) patterns of films were recorded on a Bruker AD8 ADVANCE Bruker diffractometer that was equipped with a PSD Vantec detector and a Göebel mirror to confirm their amorphous nature. Cu Kα radiation (wavelength (λ) of 1.541 Å) was used in reflection mode, with a scattering angle (Θ) range of 3–80°. A step-scanning mode was utilized for the detector with a 2Θ step size of 0.024° and a duration of 0.5 equiv per step. The apparent *d*-spacing (*d*) related to the most preferential intersegmental distance was calculated using Bragg′s law (eq 1).
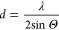
1

Density measurements (ρ) were carried out on the membranes and determined based on the Archimedes principle using a top-loading electronic XS105 Dual Range Mettler Toledo balance coupled with a density kit. Each sample was weighed six times in air and then six times in high-purity water at room temperature. The density was calculated from average weights by using eq 2.
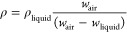
2where ρ_liquid_ is the density of water, *w*_air_ is the sample weight in air, and *w*_liquid_ is the sample weight in water.

The fractional free volume (FFV) of the membranes was estimated from density data by applying the eq 3.
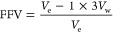
3where *V*_e_ is the specific volume and *V*_w_ is the van der Waals volume, which was calculated through molecular modeling of the polymer repeating units by applying the semiempirical Austin Model (AM1) in the Biovia Materials Studio Program.

Nitrogen and CO_2_ gas adsorption experiments of microporous (<2 nm) and mesoporous (2–50 nm) materials were performed on the polymers under isothermal conditions. All the measurements were carried out in a 3Flex Micrometrics high-performance adsorption analyzer. Samples were measured first in nitrogen at 77.3 K using an equilibration interval of 20–25 s and a degasification temperature of 180 °C overnight. After N_2_ measurements, another degasification step was done to the samples in the same conditions, and CO_2_ adsorption experiments were carried out at 273 K using an equilibration interval of 70–100s.

### Selective Ion Transport through PIM Membranes

The ion transport of alkali salt aqueous solutions across the PIM membranes was assessed by concentration-driven dialysis diffusion tests at 25 °C. The tests were carried out by using H-shaped cells consisting of two tanks, one denominated as feed and the other as permeate, interconnected, and separated by the membrane with an effective surface area of 1.53 cm^2^ (included in Supporting Information, Section 2.1). The membranes were fitted between the two tanks using phenolic screw caps (SLV 15) and PTFE sealing rings. Deionized water (15 mL) was used to fill the permeate tank, while the feed tank contained an aqueous solution (15 mL) of different salt concentrations (1, 2, and 4 M LiCl; 2 M NaCl; and 2 M KCl). Solutions in both tanks were stirred to reduce the polarization due to differences in the concentration values near the membrane. The conductivity value in the permeate tank was continuously measured every hour by using a conductivity-meter (Eutech PC 2700, Thermo Scientific) until the steady-state was reached. The conductivity–concentration calibration curve for a particular salt (LiCl, NaCl, and KCl) was used to obtain the concentration data in the permeate solution over time, and the permeability (*P* in cm^2^·s^**–**1^) was then calculated from eq 4. (More details about the diffusion tests can be found inSupporting Information, Section 2.3.)
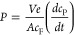
4where *V* is the solution volume of 15 mL; *C* is the salt concentration in the permeate solution (*c*_P_) and the feed solution (*c*_F_) in mol·L^–1^; *A* and *e* are the effective area (15.3 cm^2^) and the thickness of the membrane (cm), respectively; and *dc*_p_*/dt* is the steady-state rate of salt concentration in the permeate side. The determination of the alkali ion concentration (mg·L^–1^) in the permeated side of the permeability cell after 100 h was performed by inductively coupled plasma and mass spectrometry (ICP-MS) in an Agilent elemental analyzer (7900 model).

### Electrochemical Impedance Spectroscopy (EIS)

The ionic conductivity of the polymer membranes in different salt solutions was determined by electrochemical impedance spectroscopy (EIS). The EIS was performed by applying a low sinusoidal voltage of 10 mV to the working electrode. Frequencies ranged from 7 MHz to 10 mHz, with the multichannel potentiostat/galvanostat (Biologic SP-300) used at the open circuit potential. The measurements were conducted in Swagelok cells, where the polymer membrane was sandwiched between two stainless steel blocking electrodes. The temperature was controlled by placing the cell inside a Buchi glass oven (B-585 model) capped with a Teflon tap. For each of the electrode compositions, impedance plots were recorded holding the cell at each temperature for 1 h for temperature stabilization.

### In-Line ^7^Li, ^1^H, and Static ^35^Cl Solution NMR Studies of Ion Diffusion and Crossover

To study the behavior of the modified PIM-Ki-COOH in aqueous solution, in-line ion diffusion experiments and in-line crossover experiments^28−30^ were carried out using a Fourier 80 Bruker benchtop NMR (80 MHz). The schematic of the setup is shown in Figure S22, Section 3.1. In-line ^7^Li NMR experiments were performed using 0.5 M aqueous solutions of LiCl and 100 mM of Li_3_Fe(CN)_6_ to probe if the Li^+^ cations pass through the membrane. The flow cell assembly was made using a 40 μm thickness PIM-Ki-COOH membrane as ion-transport membrane, then a 25 mL tank containing the salt solution was connected to one of the chambers of the cell, and the other one and the NMR magnet were connected in-line to another tank with deuterated water D_2_O (Figure S22). Continuously, water that came out of the chamber was pumped through the magnet at 4 mL·min^–1^. The passage of ions through the membrane was monitored following the ^7^Li signal for 24 h. In addition, static ^35^Cl-NMR measurements were carried out to the resulting permeated solution to examine whether the Cl^–^ ions also permeated together Li^+^ ions.

We also carried out in-line ^1^H NMR crossover experiments to demonstrate that the micropores of the modified membranes block the passage of the redox active species used as catholyte and anolyte in RFB (Li_3_Fe(CN)_6_ and anthraquinone-2,7-disulfonic acid dilithium salt) while allowing lithium ions to pass (Supporting Information, Section 3.1, Figure S22). PIM-Ki-COOH membranes of 40 μm thick were used. In this case, water proton signal was followed by NMR because the presence of paramagnetic [Fe(CN)_6_]^3–^ anions shifts the proton signal to lower chemical shift values (Supporting Information, Section 3.3, Figure S24 A and B), and on the other hand, anthraquinone-2,7-disulfonic anion has characteristic signals in the aromatic region, which make it simpler to see if there is any crossover (Figure 9B). Li_3_Fe(CN)_6_ was used for this experiment instead of Li_4_Fe(CN)_6_ due to the Fe^3+^ paramagnetic property. In Li_3_Fe(CN)_6_, the iron center has an oxidation state of (III), resulting in a [Ar]3d5 configuration with an unpaired electron, whereas Li_4_Fe(CN)_6_ has a closed shell configuration of [Ar]3d6. The bulk magnetic susceptibility effect (BMS) induced by the presence of iron(III) directly impacts the water signal under a static magnetic field, such as the one from the NMR magnet, resulting in a shift of the signal and peak broadening (Supporting Information, Section 3.3, Figure S24A,B). Both effects can be used to detect if there is a paramagnetic ion, i.e., Fe(CN)_6_^3–^, crossover over time. Thus, in the case of crossover, the change of the chemical shift can be used to quantify the concentration of paramagnetic species. This can be achieved by using Evan’s method,^31^ which is a direct relation between the chemical shift and its dependence on the BMS of the sample, which is also proportional to the concentration of ions (see Supporting Information, Figure S25). Another reason to use the Li_3_Fe(CN)_6_ is that it has a higher solubility in water than the potassium salt.^18^ (More information about calibration conditions can be found in Supporting Information, Section 3.4.)

### Solid-State NMR Studies

^13^C and ^7^Li solid-state NMR experiments were performed on PIM-Ki-COOH films before and after a LiCl diffusion experiment to study the interactions between the carboxylic groups (COO^–^) and the Li^+^ ions. A 300 MHz Varian NMR spectrometer was used, equipped with a 4 mm Bruker probe resonant for ^13^C at 75.4 MHz and ^7^Li at 116.6 MHz. Cross-polarization magic angle spinning (CPMAS) was used to enhance the ^13^C signal. Proton decoupling using a spinal-64 sequence was used to increase the resolution. Spectral editing techniques were used to facilitate assignment; interrupted decoupling highlighting nonprotonated carbons, short contact cross-polarization (20 μs) for protonated carbons, and 2D-PASS to obtain spinning sideband free spectra. Typical RF fields were 50 kHz for both ^1^H and ^13^C, whereas spinning speeds were usually 5 kHz. ^7^Li spectra were acquired also at 5 kHz spinning speed. The PIM-Ki-COOH (65%) membrane was used in these experiments because it performed best in the diffusion and crossover experiments (more details in Supporting Information, Section 4).

### Half-Cell Experiments

The study of the properties of the PIM-Ki-COOH (65%) membrane as an ion-conducting medium with high-selectivity was performed in an aqueous half-redox flow cell at neutral pH value (Figure 10). In this symmetric cell configuration, an aqueous solution of 0.1 M Li_4_Fe(CN)_6_ acted as a catholyte, whereas the anolyte was an aqueous solution of 0.1 M Li_3_Fe(CN)_6_. Both analytes also contained 0.5 M LiCl as a supporting salt to reduce the polarization on both sides of the membrane. The electrolytes were pumped into the electrochemical cell by means of a diaphragm pump (KNF, model SIMDOS 10 FEM 1.10 S) at different flow rates (20–90 mL·min^–1^). The PIM-Ki-COOH membrane with an effective area of 5.29 cm^2^ was placed between two porous carbon electrodes (Sigracell GFA 6 EA). The assessment of the electrochemical reversibility, capacity retention, rate capability, coulombic efficiency, and cyclability of the cell at neutral pH value was carried out by using an SP-300 Potentiostat (Biologic). The cell was tested at different current densities ranging from 0.2 to 40 mA·cm^–2^ under a cutoff voltage window between 0.8 and −0.8 V. Five consecutive galvanostatic charge and discharge cycles at different current densities and flow rates were performed. Before the cycling experiments, a 2 M LiCl solution was fed into the cell with the aim of preconditioning the membrane for lithium ion transport. Ion transport across the membrane should be enough to achieve a charge balance between both sides of the cell during the polarization process.

## Results and Discussion

### Polymerization Reaction

**Figure 1:**
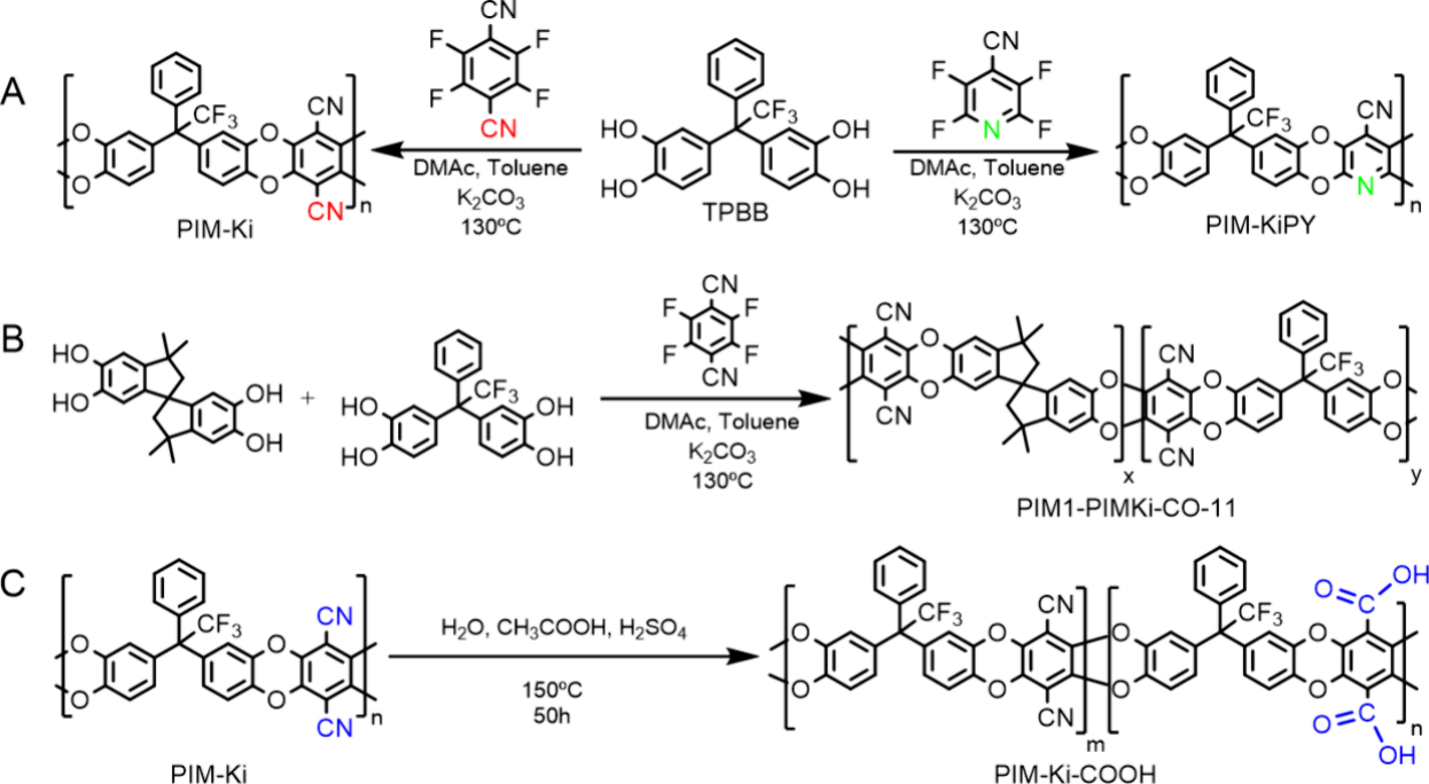
Synthetic routes: (A) PIM-Ki (left) and PIM-KiPY (right). (B) PIM1-CO-PIMKi-11 copolymer. (C) Postmodification of PIM-Ki by carboxylation reaction at different times, 50 and 150 h.

Figure 1 shows the three dibenzodioxin-based PIMs synthesized in this work (Figure 1A,B). All of them were prepared by using a modified procedure of the classical S_N_Ar polycondensation reaction employed for making these kinds of polymers.^1^ On the one hand, the temperature was reduced from 155 to 130 °C to avoid the formation of a cross-linked polymer, and on the other hand, toluene was added to the reaction to remove the water produced during the polycondensation reaction due to the formation of the water/toluene azeotrope, promoting the polymerization. All the PIMs were obtained with moderate yields (>80%) (Supporting Information, Section 1).

**Figure 2:**
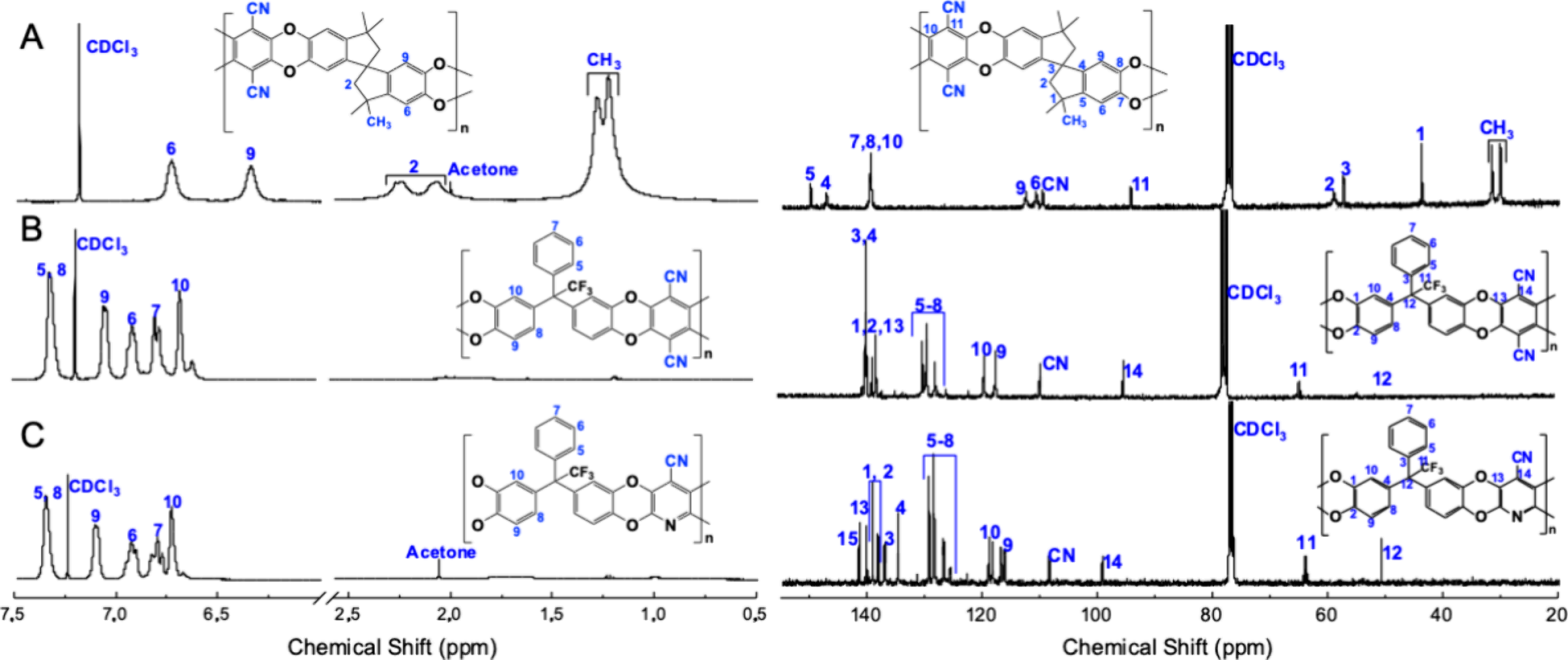
^1^H NMR spectra (left) and ^13^C NMR spectra (right) of PIM-1 (A), PIM-Ki (B), and PIM-KiPY (C) in CDCl_3_.

**Figure 3:**
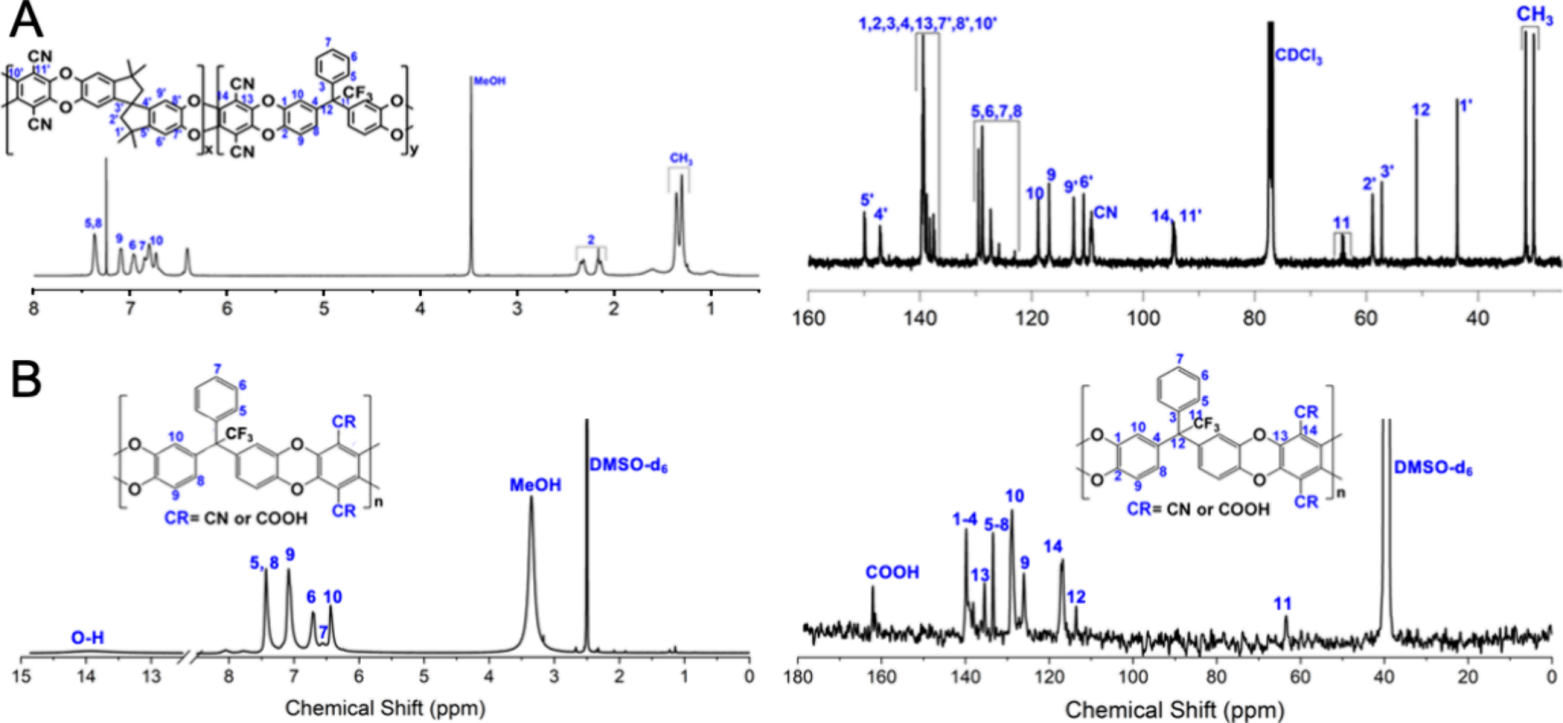
^1^H NMR spectra (left) and ^13^C NMR spectra (right) of PIM1-PIMKi-CO-11 (A) in CDCl_3_ and PIM-Ki-COOH (B) in DMSO-*d*_6_.

The chemical structure of PIM-1, PIM-Ki, PIM-KiPY, and PIM1-PIMKi-CO-11 was confirmed by ^1^H and ^13^C NMR. These polymers were soluble in a few organic solvents: CHCl_3_ and THF. The spectra of these PIMs are shown in Figures 2 and 3, where the aromatic and aliphatic protons and carbons of the polymers are assigned to their corresponding resonances (the full spectra are shown in Figures S4–S13). The ^1^H NMR spectra do not show the resonance associated with hydroxyl (OH) groups (around 9 ppm) from TTSBI and TPBB monomers, indicating that the formation of dibenzo-*p*-dioxin moieties has taken place (the TPBB OH resonance is in Figure S2, and the absence of this signal is in Figures S4 and S6). Aromatic proton peaks appear in the range of 6.5–7.5 ppm in all of the polymers. In addition, aliphatic protons corresponding to the methylene (CH_2_) and methyl (CH_3_) groups are shown at 2–2.5 and 1–1.5 ppm in the PIM-1, respectively (Figure 2A). On the other hand, the ^13^C NMR spectra have also been useful in identifying the peaks of the −CN group at 109.31 ppm, the −CF_3_ group at 63.29 ppm, and the quaternary carbons at 57 ppm (Figure 2A) and at 51 ppm in the PIM-Ki and PIM-KiPY (Figure 2B,C).

As can be expected, the ^1^H and ^13^C NMR spectra of the PIM1-PIMKi-CO-11 copolymer (Figure 3A) show characteristic peaks of both PIM-1 and PIM-Ki. The chemical structure was also confirmed from ATR-FTIR spectra of the polymers (Supporting Information, Section 1.4, Figure S15). All the polymers show absorption bands at 1606, 1508, and 1446 cm^–1^ assigned to the stretching C_ar_–C_ar_ vibrations and at 2240 cm^–1^ to the stretching C≡N vibration. Additionally, PIM-1 shows a band around 3000 cm^–1^ corresponding to the stretching aliphatic C–H vibrations of the spiro moiety and PIM-Ki at 1145 cm^–1^ associated with the stretching C–F vibrations of −CF_3_ groups. PIM-KiPY shows the same band at 1145 cm^–1^ and another band at 1630 cm^–1^ assigned to the stretching C=N vibration of the pyridine group. All the absorption bands mentioned are observed in the PIM1-PIMKi-CO-11.

### Postmodification of PIM-Ki

The compatibility between the aqueous-based analytes (i.e., redox species and/or electrolytes) with neutral pH or slightly acidic conditions and the membranes is of crucial importance for achieving their full potential when they are combined in sustainable electrochemical energy-related devices.^24,25^ For this purpose, PIM-Ki was chemically modified by an acid hydrolysis method (Figure 1C) previously reported.^27^ The hydrolysis reaction was carried out at different times: 50 and 150 h. The transformation of the −CN groups into −COOH ones changes the solubility, with PIM-Ki-COOH being soluble in aprotic moderate and strong polar solvents, such as THF, DMF, and DMSO, in which PIM-Ki was insoluble, but not in chloroform (see Supporting Information, Section 1.3).

**Figure 4:**
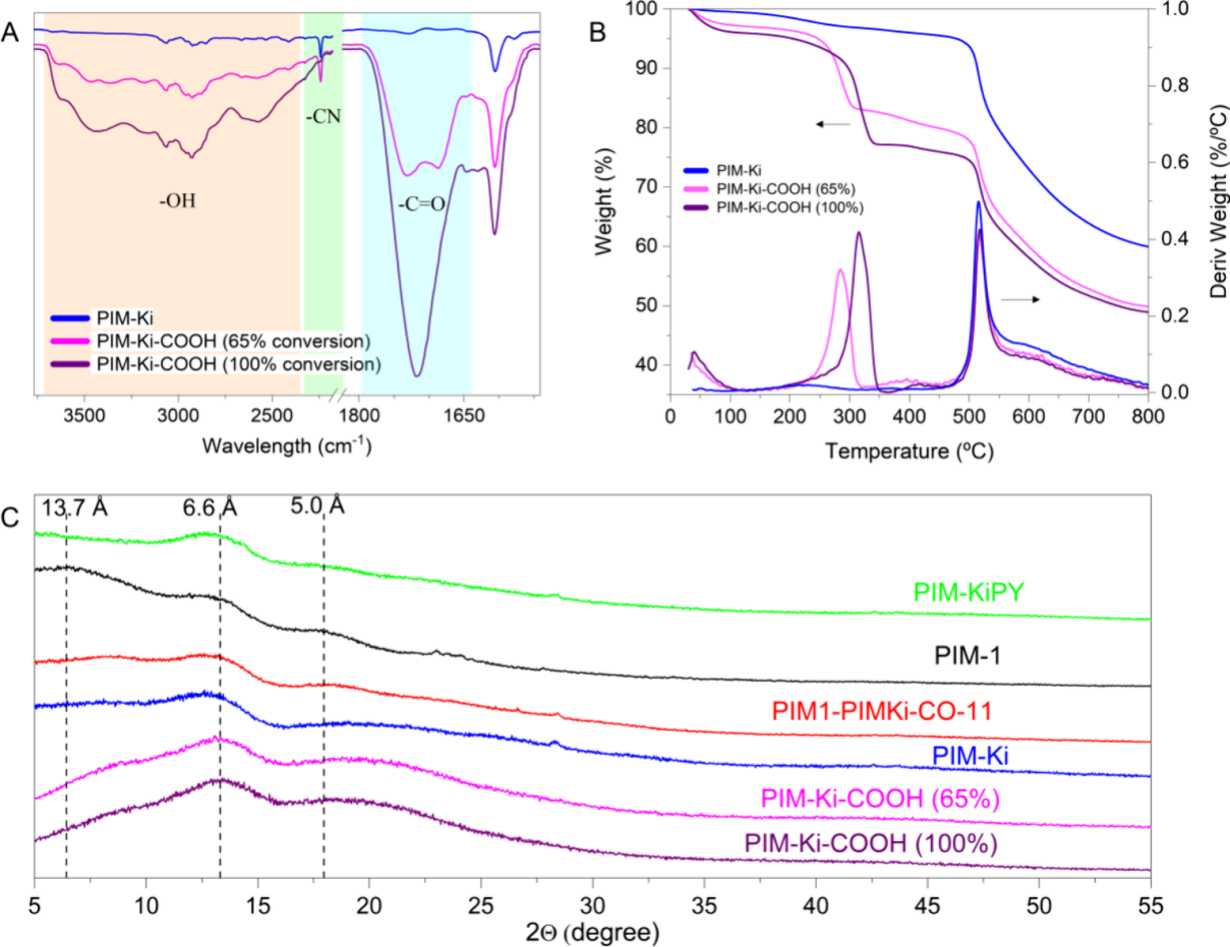
(A) Normalized ATR-FTIR spectra at the absorption band at 1446 cm^–1^ of PIM-Ki and PIM-Ki-COOHs. (B) TGA thermograms of all of the polymers. (C) Normalized WAXD patterns at the maximum at 13.3° of polymers.

^1^H and ^13^C NMR spectra of PIM-Ki-COOH are shown in Figure 3B. In this case, the signals seem to be broader than those of PIM-Ki due likely to the high water uptake of PIM-Ki-COOH, and spectra with high resolution could not be obtained; thus the −CN and −COOH groups could not be identified by NMR. However, the presence of these groups is confirmed by ATR-FTIR spectroscopy as observed in Figure 4A (the full spectrum of a carboxylated PIM-Ki is shown in Figure S15). The C=O stretching band appears at 1719 cm^–1^, and the OH stretching vibration provides a broad absorption band in the 3000–3500 cm^–1^ range. In addition, the modification progress of the −CN groups into the −COOH groups could be monitored. The spectra were previously normalized to the band at 1446 cm^–1^ (C=C), which should remain unaffected by the conversion. Concomitantly, the stretching bands of carbonyl and hydroxyl present in carboxylic acids emerge and increase progressively in intensity with the hydrolysis time, whereas the vibration band of the −CN groups decreases in intensity.

The degree of conversion of −CN groups into −COOH groups was determined by TGA analysis. The thermograms of the two carboxylated PIM-Ki-COOH are compared to that of PIM-Ki in Figure 4B. Both show two weight-loss steps: the first one associated with the decarboxylation process (below 400 °C) and the second with the general degradation of polymer, which takes place at the same temperature as the degradation process of the PIM-Ki. The conversion of the −CN groups into −COOH groups was calculated from the weight loss in the first step. Thus, conversions of 65 and 100% were achieved after 50 and 150 h of hydrolysis, respectively, relative to a theoretical weight loss of 15.6%.

The thermal stability of polymer membranes was also studied by TGA analysis. The thermograms are shown in Supporting Information, Figure S14, and the onset degradation temperature (Td) is listed in Table 1. The thermal stability of PIM-Ki and PIM-KiPY is about 30 °C superior to that of PIM-1. The Td of PIM1-PIMKi-CO-11 copolymer is closer to that of PIM-Ki (20 °C below the Td of PIM-1). The carboxylated polymers show the lowest Td values due to the decarboxylation process commented on above. All the polymers exhibit high char yields superior to 40 because they are highly aromatic.

**Table 1:** Thermal Analysis and Gas Adsorption Results for the PIMs

				gas adsorption			
	thermal analysis			N_2_ at 77 K		CO_2_ at 273 K	
sample	*T*_d_ (°C)	char yield at 800 °C (%)	FFV	*S*_BET_ (m^2^·g^–1^)	*V*_total_^b^ (cm^3^·g^–1^)	*S*_DR_^c^ (m^2^·g^–1^)	*V*_ads_^d^ (cm^3^·g^–1^ STP)
PIM-1	468	55	0.34	784	0.53	467	58
PIM-Ki	503	61	0.22	468	0.40	254	33
PIM-KiPY	497	40	0.17	370	0.30	nm^e^	nm^e^
PIM1-PIMKi-CO-11	487	63	0.28^a^	516	0.46	344	45
PIM-Ki-COOH (65%)	261	51	0.26^a^	493	0.52	301	40
PIM-Ki-COOH (100%)	271	50	0.17	213	0.22	252	34

a The values were obtained from FFVs of PIM-1, PIM-Ki, and PIM-Ki-COOH based on their composition.

b Total pore volume at *p*/*p*_0_ = 0.96.

c *S*_DR_ is the surface area determined from CO_2_ adsorption by applying the Dubinin–Radushkevich equation.

d Adsorbed CO_2_ volume at *p*/*p*_0_ = 0.03.

e Not measured.

The amorphous nature of all of the polymers was confirmed by WAXD. The normalized patterns display a broad amorphous halo with at least two well-defined maxima. The reference polymer, PIM-1, shows three maxima at 6.44, 13.3, and 18.0° (2Θ) corresponding to preferential intersegmental distances in the chain packing of 13.7, 6.6, and 5.0 Å. The contribution to the packing of the largest distance (13.7°) decreases considerably in PIM-Ki, PIM-KiPY, and PIM1-PIMKi-CO-11, indicating a higher packing density in these three polymers relative to PIM-1. On the other hand, this contribution is still lower in carboxylated polymers, whereas that of the lowest distance (6.6 Å) increases, revealing that −COOH groups could form hydrogen bonds that would lead to even higher packing density.^7,32^

The fractional free volume (FFV) of membranes was estimated based on their bulky density by applying eq 4, and the values are listed in Table 1. PIM-1 exhibits the highest FFV (0.34), confirming a lower packing density than the other polymers. The FFV decreases by 35% in PIM-Ki, by 50% in PIM-KiPY, and by 18% in PIM1-PIMKi-CO-11 relative to PIM-1. These data reveal that TPBB monomers lead to a more efficient chain packing than TTSBI, especially if combined with the TFPyCN monomer. On the other hand, the conversion of −CN into −COOH groups appears to have a smaller effect on FFV when the carboxylated polymers are compared to their precursor, PIM-Ki.

It is well-known the gas permeability in glassy polymers is related to FFV. For that, pure-gas He, CO_2_, and CH_4_ permeability coefficients of PIM-1, PIM-Ki, and PIM-KiPY were measured at 1 bar and 30 °C. Because the high-FFV polymers are susceptible to physical aging over time, the membranes obtained by casting were subjected to the same thermal treatment; i.e., they were heated gradually up to 120 °C and maintained at this temperature for 12 h prior to measurements. The permeability and CO_2_/CH_4_ selectivity data for PIM-1, PIM-Ki, and PIM-KiPY are listed in Table S2 in the Supporting Information. It is observed that the gas permeability of the membranes follows the order PIM-1> PIM-Ki > PIM-KiPY in agreement with the FFV data. In addition, all of them show higher permeability to CO_2_ than to He and the lowest permeability to CH_4_, which is the largest molecule.^33^ This behavior (PCO_2_ > PHe) appears in polymers with intrinsic microporosity.^34−36^ The permeability in both PIM-Ki and PIM-KiPY is about 70% lower for He, 80% lower for CO_2,_ and between 80 and 90% for CH_4_, respectively, relative to PIM-1. On the other hand, the membranes are subjected to permeability–selectivity trade-off;^33^ i.e., the increase in CO_2_ permeability is accompanied by a decrease in CO_2_/CH_4_ selectivity. Thus, the selectivity increases by 150% higher for PIM-Ki and by 170% for PIM-KiPy relative to that of PIM1. These findings indicate that chain packing of PIM-1, derived from TTSBI, leads to the formation of a higher amount of free volume elements (or voids) than those in the other two polymers.

**Figure 5:**
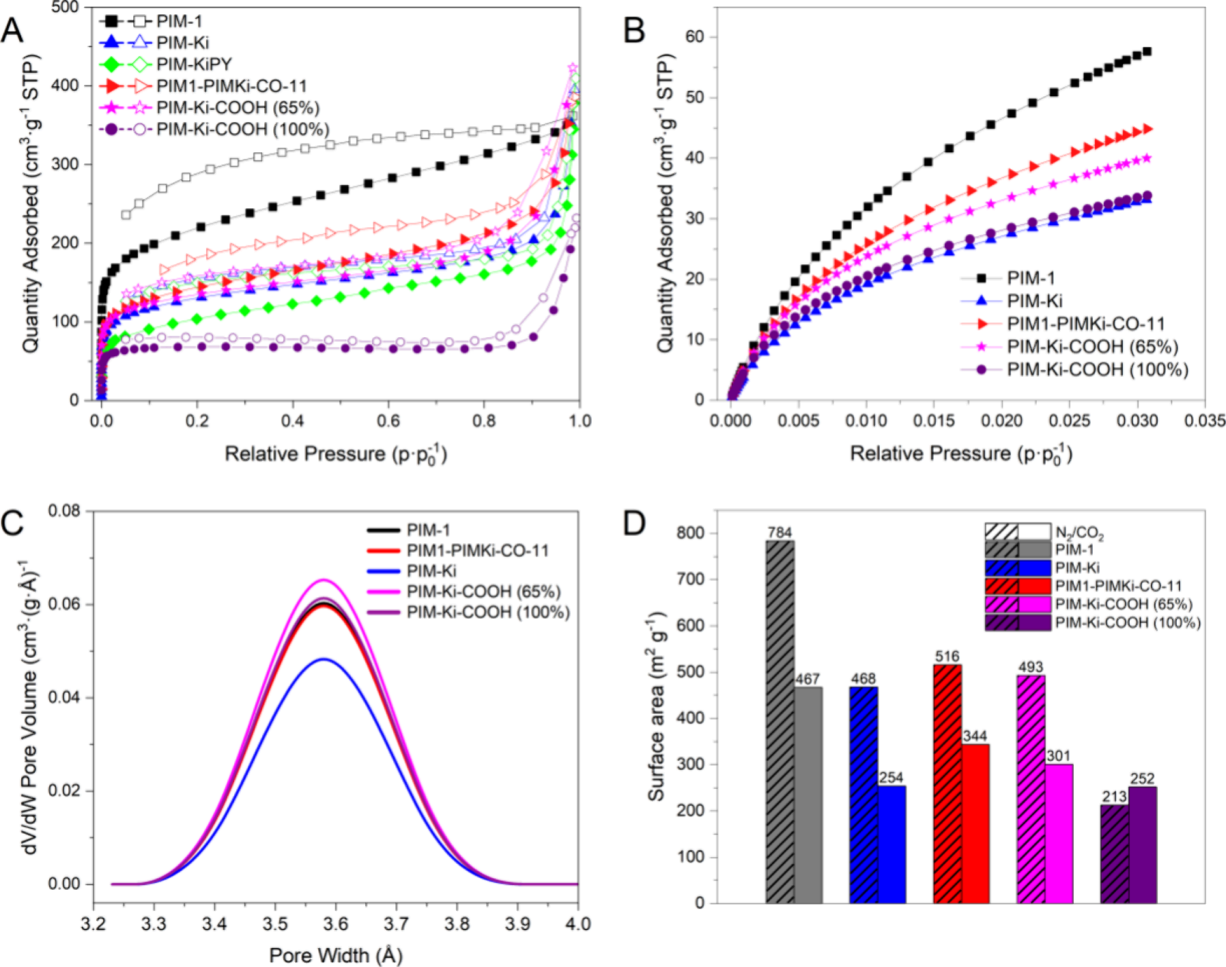
(A) Low-pressure N_2_ adsorption/desorption isotherms at 77 K and (B) low-pressure CO_2_ adsorption isotherms at 273 K of the PIMs and modified PIM-Ki-COOH (65%). (C) Pore size distribution from CO_2_ isotherms obtained from NLDFT analysis. (D) Surface areas of the PIMs: *S*_BET_ and *S*_DR_ were obtained from adsorption isotherms of N_2_ and CO_2_, respectively.

The porosity of polymers was investigated by low-pressure adsorption/desorption N_2_ isotherms at 77 K. All of the isotherms are compared in Figure 5A. PIM-1, PIM-Ki and PIM-KiPY show a sharp uptake at low relative pressures (*p*/*p*_0_ < 0.01) in the adsorption branch, revealing microporosity. However, unlike the microporous materials that exhibit type I isotherms,^37^ the adsorption branch in these polymers does not reach a plateau when the relative pressure increases, which can be attributed to swelling phenomena.^38^ In addition, there is a considerable uptake increase above *p*/*p*_0_ = 0.8 in the adsorption branch of PIM-Ki and PIM-KiPY, which is usually associated with the presence in the material of very large mesopores and macropores. The desorption branches of the three polymers show a hysteresis down to low relative pressures, which is typical of microporous materials.^39^ The volume difference between the adsorption and desorption branches is higher for PIM-1 than for PIM-Ki and PIM-KiPY. As a result, the additional volume at *p*/*p*_0_ = 0.2 in the desorption branch is 70 cm^3^·g^–1^ for PIM-1, 45 cm^3^·g^–1^ for PIM-KiPY, and 24 cm^3^·g^–1^ for PIM-Ki, suggesting a higher molecular rigidity in PIM-Ki. The adsorption/desorption isotherms of PIM1-PIMKi-CO-11 are like those of PIM-Ki, but the additional volume at *p*/*p*_0_ = 0.2 in the desorption branch is higher (40 cm^3^·g^–1^). As to carboxylated polymers, PIM-Ki-COOH (65%) shows the same behavior as PIM-Ki, whereas the hysteresis of PIM-Ki-COOH (100%) practically disappears below *p*/*p*_0_ = 0.8, which confirms the formation of hydrogen bonds leading to a higher rigidity and packing density relative to PIM-Ki.

The specific area surface (*S*_BET_) and total volume (*V*_total_) were calculated from the N_2_ adsorption branch of polymers, and the values are listed in Table 1. The *S*_BET_ of nonmodified polymers depends on the starting monomers. Thus, *S*_BET_ (m^2^ g^–1^) follows the following order: PIM-1 (784) > PIM1-PIMKi-CO-11 (516) > PIM-Ki (468) > PIM-KiPY (370). On the other hand, the total functionalization of PIM-Ki to PIM-Ki-COOH (100%) results in a 55% reduction in *S*_BET_, as expected. In contrast, the *S*_BET_ of PIM-Ki-COOH (65%) is only 5% higher than that of PIM-Ki.^27^ This finding is unexpected. However, it is interesting that the hydrophilicity of PIM-Ki increased without losing *S*_BET_.

Low-pressure adsorption isotherms using CO_2_ were performed at 273 K, as seen in Figure 5B. The narrow pore size distribution (PSD) of nonmodified and modified polymers were determined from these isotherms via the NLDFT model on carbon-slit pores, which are shown in Figure 5C. The PIM1, PIM-Ki, and PIM1-PIMKi-CO-11 display similar PSD with narrow micropores (<7 Å) between 3.2 and 3.9 Å (maximum at around 3.6 Å), which cannot be characterized by N_2_ at 77 K. However, PIM-Ki presents a lower amount of narrow micropores than the other two. On the other hand, it seems the functionalization of PIM-Ki creates more of these narrow micropores, and they show similar PSD to those of PIM1 or PIM1-PIMKi-CO-11, even somewhat superior in the case of PIM-Ki-COOH (65%). The surface area (S_DR_) was calculated from the isotherms by applying the Dubinin–Radushkevich equation. S_DR_ and *S*_BET_ values follow the same trend, as seen in Figure 5D. From these data, it can be concluded that 65% modification of PIM-Ki seems to lead to the formation of micropore sizes below 0.8 nm, increasing the CO_2_ adsorption relative to that of PIM-Ki.

### Ion Transport Studies in Aqueous Electrolytes

**Figure 6:**
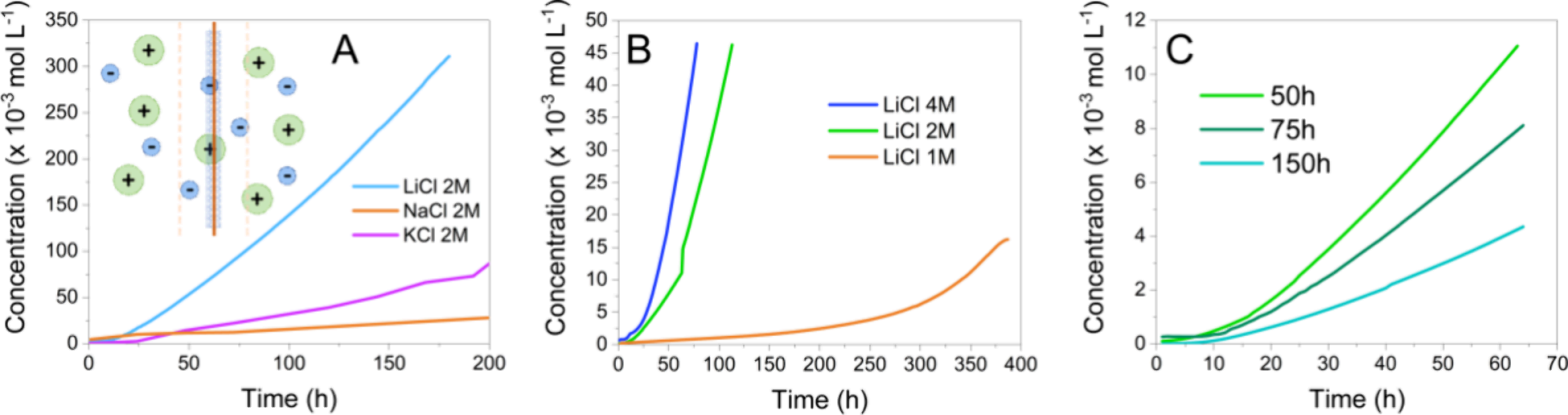
(A) Permeability experiments by using a 2 M concentration of different monovalent alkaline metal salts (LiCl, NaCl, KCl) as feed aqueous solutions. (B) Permeability experiments by using different concentrations of LiCl salt as feed aqueous solutions. (C) Permeability experiments in 2 M LiCl by using membranes with different functionalization reaction times, which mean different ratios of modification for −CN groups.

Owing to the almost negligible monovalent alkali metal ion transport across the unmodified PIM-Ki membranes (Figure S18), the permeability studies were performed on those membranes for which cyanide groups were chemically transformed into carboxylic groups to improve the affinity of the membranes in aqueous media and then promote the passage of the ions through them. Permeability experiments were performed by using a 2 M concentration of different monovalent alkaline metal salts (LiCl, NaCl, and KCl) as feed aqueous solutions (the 2 M salt solution was the concentration chosen for the permeability tests because it is closer to those concentration values that are used in an electrochemical device like a redox flow battery).^26^ The PIM showing 65% of conversion of cyanide to carboxylic groups (PIM-Ki-COOH (65%) evidenced a higher selectivity toward the ionic species of the lithium chloride salt than for their sodium and potassium counterparts as shown in Figure 6A. Despite the subtle size difference between hydrated radius of Li^+^ (0.382 nm), Na^+^ (0.358 nm), and K^+^ (0.331 nm ions),^40^ the transmembrane flux of lithium ions displayed a higher rate, accomplishing permeability values of 2.4 × 10^–9^ cm^2^·s^–1^ compared to the other monovalent alkali metal cations analyzed (NaCl 2M, 7.38 × 10^–10^ cm^2^·s^–1^; KCl 2M, 1.05 × 10^–9^ cm^2^·s^–1^). We propose that the lithium transport is enhanced because the small–small ion pairs like Li^+^ and Cl^–^ associate stronger in water than the other alkali metals with larger radii such as Na^+^ and K^+^ ions.^41^ The strong electrostatics might provoke a smaller distance between the ions along with the stabilization of the water solvent network surrounding the contact ion pair and then promote the selective transport of lithium ions through the hydrophilic PIM-Ki-COOH (65%) membranes mainly by size sieving.

To evaluate the influence of the lithium salt concentration on the ion diffusion values, a series of aqueous solutions of LiCl with 1, 2, and 4 M concentrations in the feed tank were used. Interestingly, the transport of lithium ions across the membrane seems to be promoted after an initial stage where probably channels inside the porous membrane were preconditioned for the ion diffusion for all the concentrations.^42^ As expected, the higher the concentration of the feed solution is, the higher is the ion transport flux as is also shown in Figure 6B. The PIM-Ki-COOH membranes achieve permeability values up to 2.4 × 10^–9^ and 1.4 × 10^–8^ cm^2^·s^–1^ for the 2 and 4 M LiCl feed solution, respectively. According to these permeability results, PIM-Ki-COOH (65%) membranes show high LiCl permeabilities in near-neutral aqueous solutions, which are comparable to those of Nafion 212 or Nafion 117 with all values^43^ in the order of 10^–7^ cm^2^·s^–1^.

Membranes with different functionalization times were also tested (PIM-Ki-COOH-50h (65%), PIMKi-COOH-75h (85%), PIMKi-COOH-150h (100%)) as shown in Figure 6C. The membranes modified during 75 and 150 h showed permeability values of 4.8 × 10^–12^ and 4.6 × 10^–12^ cm^2^·s^–1^, respectively, significantly lower compared to that of the membrane chemically modified for 50 h, which corresponds to the one modified 65%. This drop in permeability is mostly due to the extended conversion of the cyanide groups into carboxylic acid reaction of the PIM-Kis leading to a probable reduction of the intrinsic microporosity of the polymer. The permeability values correlate well with the textural properties of PIM-Kis measured by gas sorption.

The concentration of the alkali ions (Li, Na, and K) on the permeated side was also measured by direct methods such as inductively coupled plasma spectrometry (ICP-MS) after 100 h of permeation across the PIM-Ki-COOH (65%) membrane because it showed the best performance in the permeability experiments (Figure 6C). The results derived from the elemental analysis by ICP-MS clearly confirm the high selectivity of our microporous membrane toward Li (1934 ± 10 mg·L^–1^) ions compared to Na (30 ± 0.5 mg·L^–1^) and K (24.7 ± 0.4 mg·L^–1^). Li/Na and Li/K selectivity ratio values >60 were evidenced from the concentration values of the permeated aqueous solutions.

Nyquist plots were also obtained from electrochemical impedance spectroscopy (EIS) measurements and are depicted in Figure S21, displaying the curves associated with the microporous polymer membrane chemically modified for 50 h (65% modification) and different aqueous electrolytes based on LiCl, NaCl, and KCl with a 2 M concentration. Because the configuration of the symmetric Swagelok cells only differs in the nature of the aqueous electrolyte, the electrical series resistance calculated from the intersection on the real axis of the Nyquist plot in the high frequency region primarily reflects the bulk membrane conductivity. It is evident that the measurement in the presence of an aqueous solution containing LiCl or KCl salt did not exhibit any Nyquist semicircle, indicating a significantly more efficient charge transfer process at the membrane interface than in the presence of NaCl salt in the electrolyte as shown in Figure S21A. The Nyquist plots were interpreted using a circuit model described by the equations inserted in the legend of each graph in Figure S21B,C. The conductivity’s dependence on temperature is shown in Figure S21D. The ionic conductivity values show a higher conductivity for the aqueous electrolytes based on LiCl than for their analogs based on NaCl and KCl. The ionic conductivity values at 30 °C for LiCl were up to 2.5 × 10^–4^ S·cm^–1^, whereas those based on NaCl and KCl were 7.4 × 10^–5^ and 6.7 × 10^–5^ S·cm^–1^, respectively. Therefore, we can conclude that the presence of LiCl salt ions solvated by water offers a less resistive surface for ion diffusion in the membrane chemically modified for 50 h and containing about 65% of carboxylic groups, consistent with the permeability results. A comparison between PIMs’ and other materials’ permeability properties^43^ is shown in Table S3.

**Figure 7:**
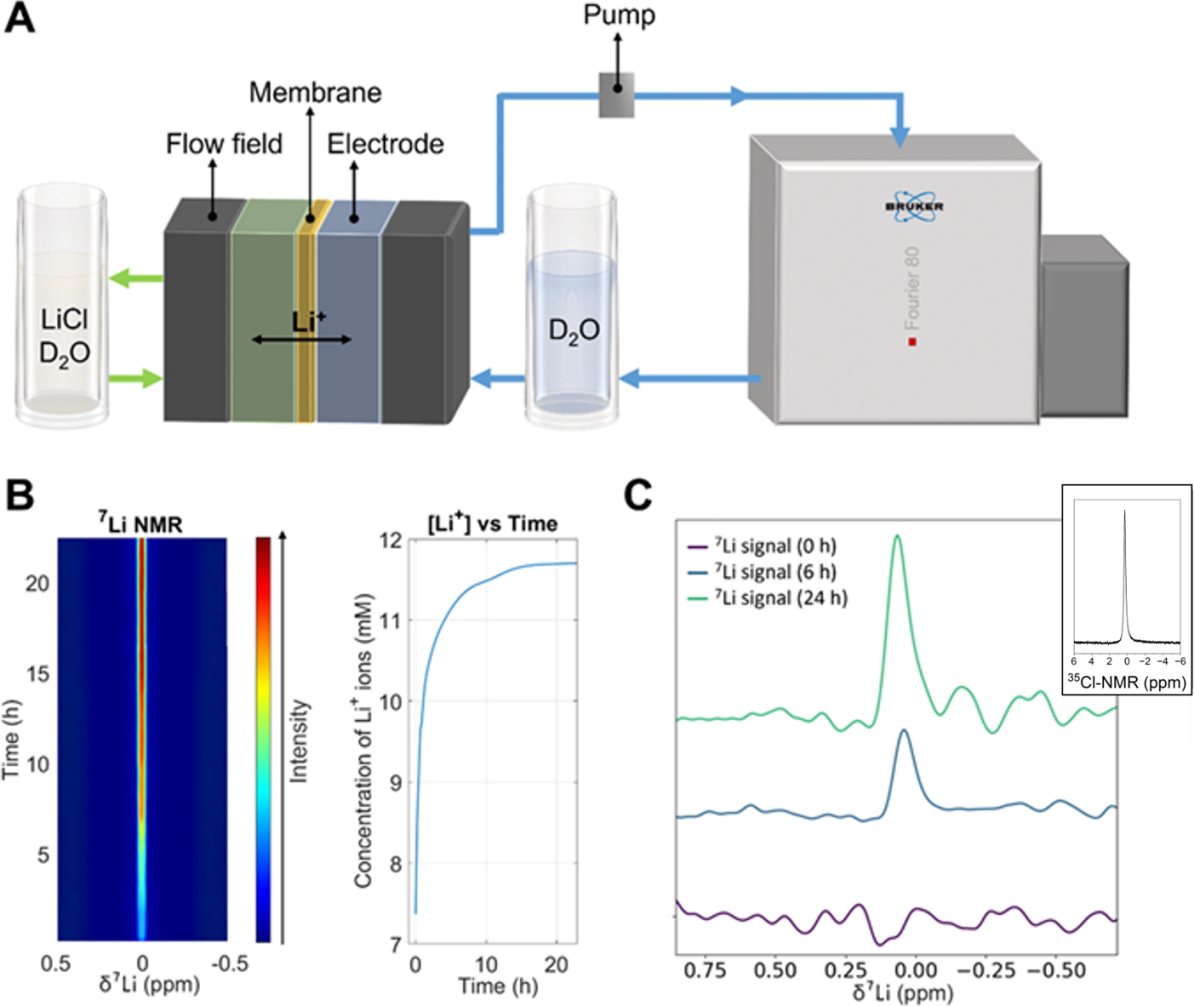
(A) Scheme of the benchtop in-line NMR setup (80 MHz Bruker) using LiCl as the electrolyte. (B) Pseudo-2D ^7^Li NMR spectrum as a function of time during the flow experiment. (C) Extracted individual ^7^Li NMR spectrum at various times during the flow experiment: start of the experiment, after 6 h, and after 24 h. Inset: ^35^Cl NMR spectrum (500 MHz, Bruker) of an aliquot from the permeate tank at the end of the flow experiment.

In-line ^7^Li NMR experiments of the PIM-Ki-COOH (65%) membrane were carried out to directly follow the lithium-ion signal after passing the membrane instead of measuring the diffusion via conductivity. Figure 7A shows the experimental set up for the in-line NMR diffusion experiments. This system comprises a flow cell connected to two tanks: one containing a 0.5 M LiCl solution and the other designated as the permeate tank where lithium and chlorine ions will diffuse. The latter is connected to a benchtop NMR flow-through apparatus that will continuously measure the ^7^Li signal of the crossed lithium ions as a function of time. The increasing intensity is shown in a pseudo-2D NMR spectrum in Figure 7B. At the start of the experiment, no ^7^Li signal is observed (see Figure 7C), but as the experiment progresses, the signal increases until it stabilizes due to the concentration of the ions on one side of the membrane and the other side of the membrane becoming equal. Figure 7C (inset) also shows the ex situ ^35^Cl NMR spectrum of an aliquot from the permeation tank after the experiment, probing the presence of Cl^–^ ions in the permeate tank and thus proving that both ions successfully pass through the chemically modified PIM-Ki-COOH (65%) membrane while flowing. This fact reinforces our hypothesis founded on the most probable formation of ion pairs between the Li^+^ and Cl^–^ ions in water to explain the permeation trend observed for the metal alkali ions.^41^

**Figure 8:**
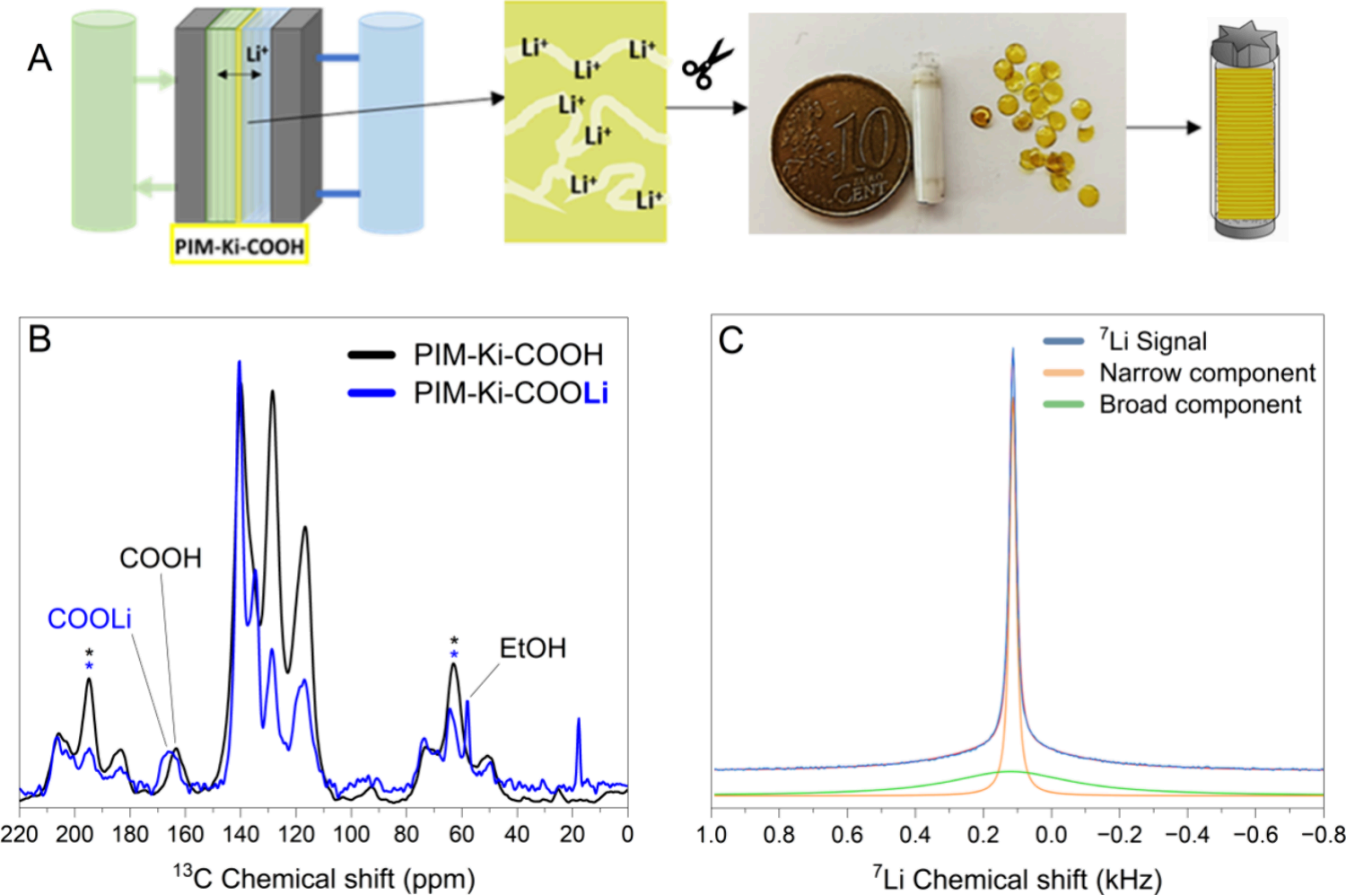
(A) Scheme of the sample preparation for the ssNMR analysis. (B) ^13^C-CPMAS-ssNMR spectra of PIM-Ki-COOH (65%) before and after the lithium diffusion experiment. (C) ^7^Li-MAS-ssNMR spectrum of the same sample deconvoluted to narrow and broad components. A 300 MHz Bruker spectrometer was used.

From these results, we propose that the lithium transport might be dominated by the size exclusion mechanism rather than a Donnan exclusion effect because the carboxylic groups of PIM-Ki-COOH (65%) (p*K*_a_ = 4) could not be fully deprotonated to produce the carboxylate ion at a neutral or close to neutral pH value. Besides, as Na and K cations diffuse much slower than lithium ones, size exclusion theory is even more probable because their ion pairs with chlorine are bigger than the one for lithium, which should end in a lower ion permeability, as we have shown in Figure 6A. However, because of the closer interactions of Li^+^ ions with the carboxylate/carboxylic moieties inside the subnanometer cages,^21^ these electrostatic interactions should also play a role, although in less extension, in the enhancement of the transport throughout the membrane. To shed light on these interactions, magic angle spinning (MAS) ^13^C solid-state (ss) NMR experiments were carried out on the PIM-Ki-COOH (65%) membrane before and after a lithium diffusion experiment. Carboxylic acids from the polymer chain can interact with lithium and will have an influence on the NMR signal.^8^ MAS ssNMR is an ideal method to study this interaction because other solution-based methods might imply dissolving the polymer, which would take the lithium off inside the porous structure of the polymeric membrane. Small 4 mm disks of the polymers were cut and stacked inside 4 mm rotors and tested in a 300 MHz Varian Magnet with a Bruker probe (Figure 8A). Cross-polarization MAS (CPMAS) from ^1^H and ^13^C was performed. Figure 8B shows ^13^C-ssNMR spectra of both membrane samples before and after lithium transport across the membrane. In the ssNMR spectrum of the pristine PIM-Ki-COOH sample, one can clearly observe the carboxylic carbon at 163 ppm (see the full spectra in Supporting Information, Section 4, Figure S26). The sample that has undergone the lithium diffusion procedure shows differences: peak intensities have drastically changed, which are most likely due to changes in polymer chain dynamics that will affect cross-polarization efficiencies. More importantly, the carboxylic carbon has shifted to 167 ppm, which could indicate coordination to Li. In the ^7^Li spectrum of the PIM-Ki-COOLi (Figure 8C), it is possible to see two Lorentzian components: a broad one of 440 Hz width (55%) and a narrow 21 Hz (more mobile) (45%). This result strongly suggests that there are Li ions present in the pores (narrow component) as well as Li ions that are less mobile, associated with the carboxyl groups. A single ^7^Li longitudinal relaxation time *T*_1_ of 0.4 s suggests that these ions are in constant exchange.

We further assessed the molecular sieving properties of our chemically modified PIM membrane by using in-line ^1^H NMR experiments. For that, two redox-active species including Li_3_Fe(CN)_6_ (hydration diameter 0.95 nm) and anthraquinone-2,7-disulfonic acid dilithium salt (hydration diameter 0.78 nm)^18^ were chosen in combination with LiCl salt as a solute of the aqueous feeding solution to perform the crossover experiments. The latter was also added as a salt support to mimic the composition of a redox electrolyte that could be found in electrochemical systems, such as a flow battery, so the impact of the properties of the PIM membrane on the electrochemical performance of a redox electrolyte can be assessed.

**Figure 9:**
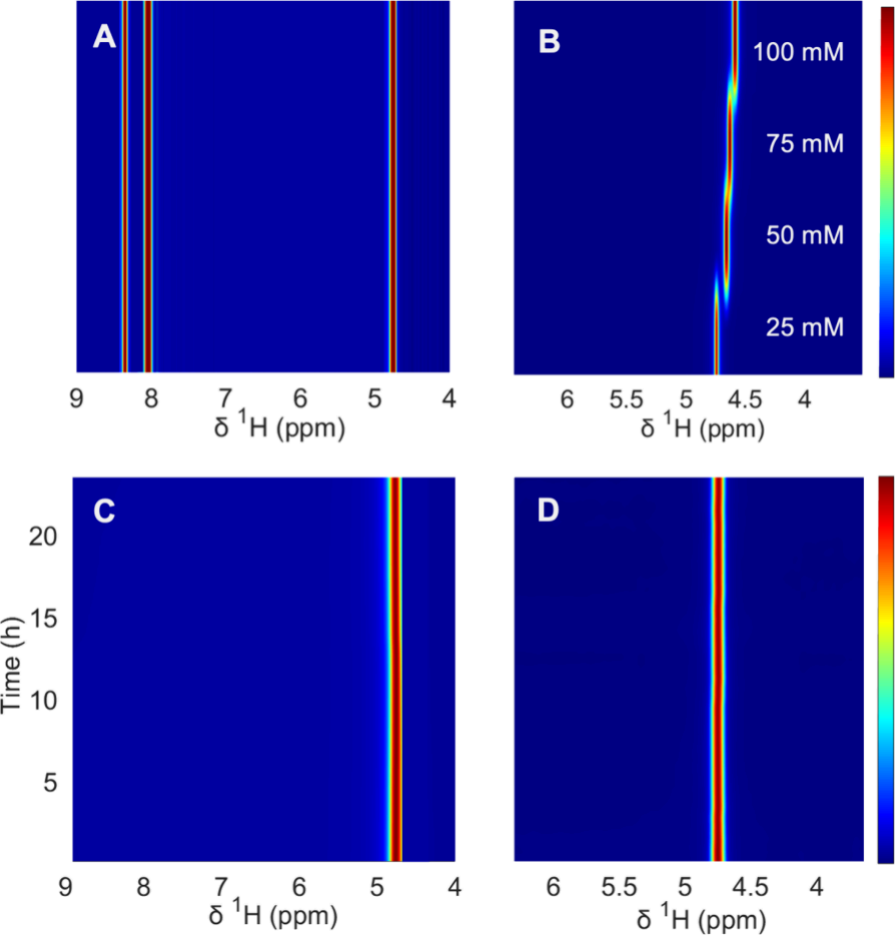
^1^H NMR spectra of Li_3_Fe(CN)_6_ and anthraquinone-2,7-disulfonic acid dilithium salt dissolved in D_2_O. (A) Contour plot generated from a single spectrum of of anthraquinone-2,7-disulfonic acid dilithium salt. (B) Contour plot generated from four Li_3_Fe(CN)_6_ solutions at different concentrations (25, 50, 75, and 100 mM). Note that we chose to represent the resulting spectra as contour plots to aid the comparison between the actual in-line experiments. This was achieved by stacking a single spectrum of the same sample tube over 10 times. (C) In-line pseudo-2D experiment of anthraquinone-2,7-disulfonic acid dilithium solution as a function of time. (D) In-line pseudo-2D experiment of Li_3_Fe(CN)_6_ solution as a function of time. Both panels C and D were acquired using PIM-Ki-COOH (65%) as the cell’s membrane.

Because the feeding solution into the benchtop NMR instrument only contains LiCl and deuterium oxide, any species crossing over from the other reservoir would be evidently seen on the ^1^H NMR spectra. To confirm this, prior to the crossover experiments, we performed simple NMR measurements in 5 mm NMR tubes for both redox electroactive analytes Li_3_Fe(CN)_6_ and anthraquinone-2,7-disulfonic acid dilithium salt. Figure 9A shows the characteristic proton signals for the anthraquinone solution, whereas Figure 9B shows how the proton signal of water in the presence of [Fe(CN)_6_]^3–^ shifted to lower chemical shifts at increasing concentrations due to the BMS effect. We also successfully assessed the influence of the salt under flow in a crossover experiment (Supporting Information, Section 3.3).

Experiments using the PIM-Ki-COOH (65%) membrane showed no crossover for any of the redox species, evidenced by the lack of extra resonances in the organic region as time progressed (Figure 9C) when the anthraquinone solution. Similarly, for the iron salt, the stability of the proton resonance from the deuterated solvent over time suggests that there is no crossover of the paramagnetic ions (Figure 9D). A more detailed discussion about the chemical shift for this last experiment has been published in the previous literature^30,44^ and is briefly explained in the Supporting Information, Figure S25.

Therefore, this membrane can successfully block the redox species while allowing lithium ions to selectively pass, as it was determined by the in-line measurements. The results confirm that a certain percentage of modified groups in the polymer membrane would be enough to allow selective transport of the alkali ions of the salt support needed to balance the charge at both sides of the membrane in an electrochemical cell while avoiding the crossover of the positive and negative electrolytes.

### Flow Experiments

**Figure 10:**
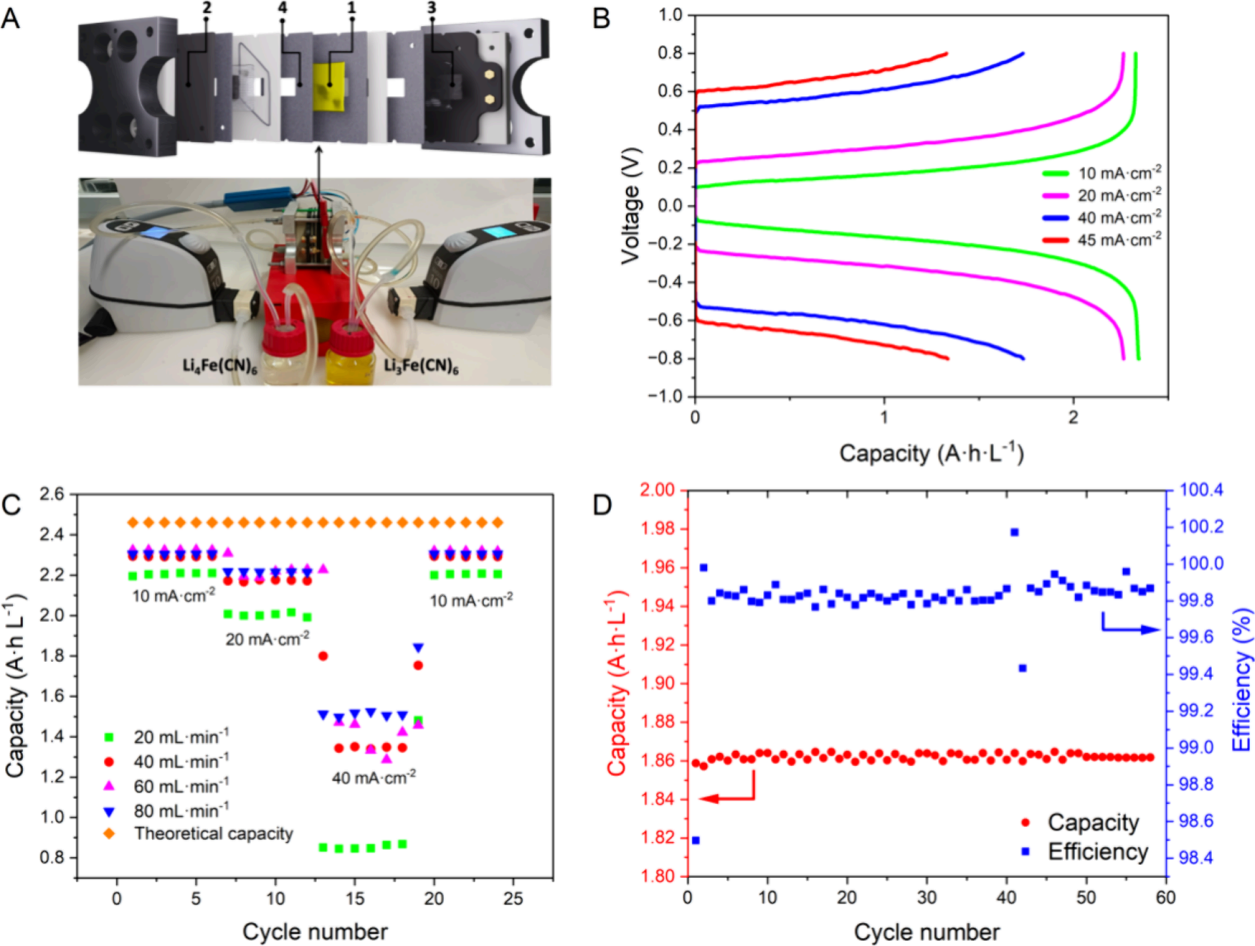
(A) Setup for the half-cell redox flow experiments using 100 mM of lithium ferro/ferricyanide and 0.5 M of LiCl as salt support (1: PIM membrane; 2: graphite current collector; 3: carbon felt; 4: rubber). (B) Representative of the cell voltage vs galvanostatic curves at different current densities at a flow rate of 60 mL·min^–1^. (C) Study of the capacity versus cycling number at different current densities and flow rates. (D) Capacity and Coulombic efficiency of the hybrid redox flow battery over 50 cycles performed at a current density of 10 mA·cm^–2^ at room temperature.

The setup for the tests carried out on the half-redox flow cell is shown in Figure 10A. This study principally allowed knowing the maximum achievable specific capacity of the electroactive species by using the new PIM membranes developed in this work. The polymer membrane was preconditioned with LiCl as the salt support during 24 h before to carry out the measurements in the presence of the Li_4_Fe(CN)_6_/Li_3_Fe(CN)_6_ redox couple. The experiments were performed at different current densities and flow rates to find the optimal electrochemical cycling conditions. Figure 10B shows that at current density values of 10 and 20 mA cm^–2^, the experimental specific capacity values achieved were near the theoretical one (115 mA·h·g^–1^) for a flow rate of 60 mL·min^–1^. A significant increase in the polarization between the oxidation and reduction processes along with a pronounce capacity decay down to 80 mA·h·g^–1^ was observed at a current density of 40 mA·cm^–2^. Figure 10C shows the rate capability of the symmetric half-cell cycled at different current densities and flow rates. High stability and capacity retention were shown after five consecutive cycles at different rates, achieving maximum values of 109 mA·h·g^–1^ representing 95% of the full cell performance. It should be also noted that the cell capacity recovers after operating under the most unfavorable conditions (40 mA·cm^–2^), proving that the polymer membranes were stable during the whole cycling experiment (Figure 10C). To avoid capacity losses and reduce the polarization, a flow rate of 60 mL·min^–1^ seems the optimum for the study of the rate capacity. The flow cell can be safely charged and discharged at a constant current (Figure 10D). A maximal capacity of 87 mA·h·g^–1^ was reached. The following charge and discharge cycles showed how the charge storage capacity decreases, although the efficiency reached values of approximately 100%. The charge–discharge curves at a current density of 10 mA·cm^–2^ are included in Supporting Information, Section 5, Figure S27. A comparison between a Nafion 117 membrane^26^ and PIM-Ki-COOH (65%) membrane is also included in Table S4.

## Conclusions

We have successfully synthesized a novel family of PIMs (PIM-Kis), chemically modified to obtain materials suitable for sustainable applications in aqueous media. A complete characterization reveals that TPBB monomer leads to a more efficient packing of polymer chains than the TTSBI one, particularly if combined with TFPyCN monomer. This fact was also confirmed by gas permeability measurements where TPBB-based membranes evidenced a high selectivity CO_2_/CH_4_ compared to their PIM-1 analogue (TTSBI-TFTPN) that was used as a benchmark membrane material.

The conversion of −CN into −COOH groups appears to have a smaller effect on FFV when the PIM-Ki-COOH polymers are compared to their precursor, PIM-Ki. However, the ability to form hydrogen bonds of the COOH groups enhances their selectivity toward lithium ions in aqueous solutions. In-line bench NMR experiments and MAS ss-NMR have fully confirmed the Li^+^ selectivity of the PIM-Ki-COOH (65%) membranes. The interaction of this ion with the polymer during the transport process leads to its selection for implementation in a symmetric redox flow cell. It has been confirmed that the crossover of iron-based redox species has been fully mitigated, and no capacity losses were observed during galvanostatic cycling at 10 mA·cm^–2^.

In summary, the PIM-Kis membranes with Li^+^ enhanced transport properties would render them suitable for use in redox flow batteries or as a protective electrode interlayer in lithium batteries and also for other electrochemical devices in which Li^+^ is the working ion, such as a salt support, or is one of the chemical species of interest, such as in a separation process.^45^

## Supporting Information Available

Experimental methods of synthesis and characterization of monomer and polymers, membrane preparation procedure, solvent solubility tests, ion transport through PIM membranes, conductivity concentration calibration plots, in-line NMR diffusion and crossover experiments, MAS solid-state NMR, and half-cell experiments (PDF)
