# Supplementary material for: Dibenzodioxin-Based Polymers of Intrinsic Microporosity with Enhanced Transport Properties for Lithium Ions in Aqueous Media
Source: Macromolecules. 2024 Sep 27;57(19):9442–56. doi: 10.1021/acs.macromol.4c01243 (PMC11468783; doi:10.1021/acs.macromol.4c01243)
Supplement: Supplementary file 1 — ma4c01243_si_001.pdf [file ma4c01243_si_001.pdf]

Supporting information

Dibenzodioxin-based polymers of intrinsic microporosity with enhanced transport properties for lithium ions in aqueous media

Juan Carlos Martínez-López,[a] Marta Santos Rodríguez,[a] Víctor Oliver Cuenca,[a] Giu Silva Testa,[b] Ernst van Eck,[b] Evan Wenbo Zhao,[b] Ángel E. Lozano,[a] Cristina Álvarez,[a] Javier Carretero-González[a,*]

[a]Institute of Polymer Science and Technology, ICTP, CSIC
C/ Juan de la Cierva, 3, 28006, Madrid, Spain
[b]Magnetic Resonance Research Center, Institute for Molecules and Materials, Radboud University, 6525 AJ Nijmegen, the Netherlands

***Corresponding author. E-mail: jcarretero@ictp.csic.es**

Section 1. Synthesis and characterization of monomer and polymers

1.1 Monomer synthesis. In this study, we use the reported method[1] with some variations in the purification steps to synthesize the 4,4'-(2,2,2-trifluoro-1-phenylethane-1,1-diyl)-bis(benzene-1,2-diol) as it is shown in Figure S1. Liming Tao et al. reported the synthesis of fluorinated bisphenols by the condensation reaction between fluorinated ketones and bisphenols catalyzed by trifluoromethanesulfonic acid, TFMS. However, as far as we know, this monomer has not been used previously to synthesize PIMs.

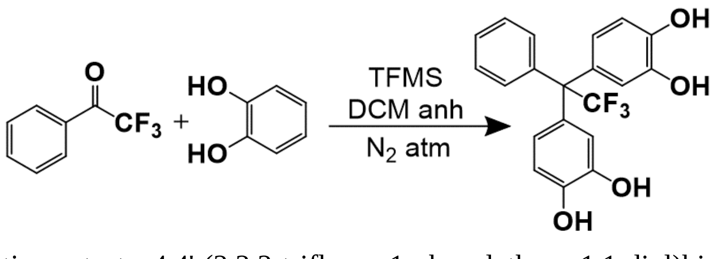

Figure S1. Synthetic route to 4,4'-(2,2,2-trifluoro-1-phenylethane-1,1-diyl)bis(benzene-1,2-diol) (TPBB) monomer.

Preparation of 4,4'-(2,2,2-trifluoro-1-phenylethane-1,1-diyl)bis(benzene-1,2-diol) or TPBB. The employed synthetic procedure was previously described by Tao.[2] In a 250 mL three-necked round-bottom flask equipped with a magnetic stirrer, N_2 inlet and a dropping funnel, pyrocatechol

(2.52 g, 0.0229 mol), 2,2,2-trifluoroacetophenone (2 g, 0.0115 mol) and anhydrous dichloromethane (100 mL) were added. While stirring, TFMS (0.858 g, 0.0057 mol) was added dropwise and the reaction color changed from white to a dark-red tone. The reaction was left stirring for 5 hours until the formation of two phases, a transparent one and a red one. After attempting the reaction, some difficulties were encountered in adhering to the reported purification steps due to the viscous nature of the product. Filtering proved to be impractical, leading to the decision to strip off the reaction solvent (dichloromethane, DCM) and introduce water into the flask to induce precipitation of the white solid from a red viscous slurry. Subsequently, filtration was performed, followed by washing with DCM and cold water. Challenges also came out during the recrystallization step due to the high solubility of the product in ethanol. Consequently, it was necessary to reprecipitate the product in a non-solvent (distilled water) and repeat the filtration process to obtain the pure white product.

The white powder obtained was dried under vacuum (80ºC) and characterized by ^{1}H and ^{13}C-NMR (Figures S2 and S3). Yield: 76%.

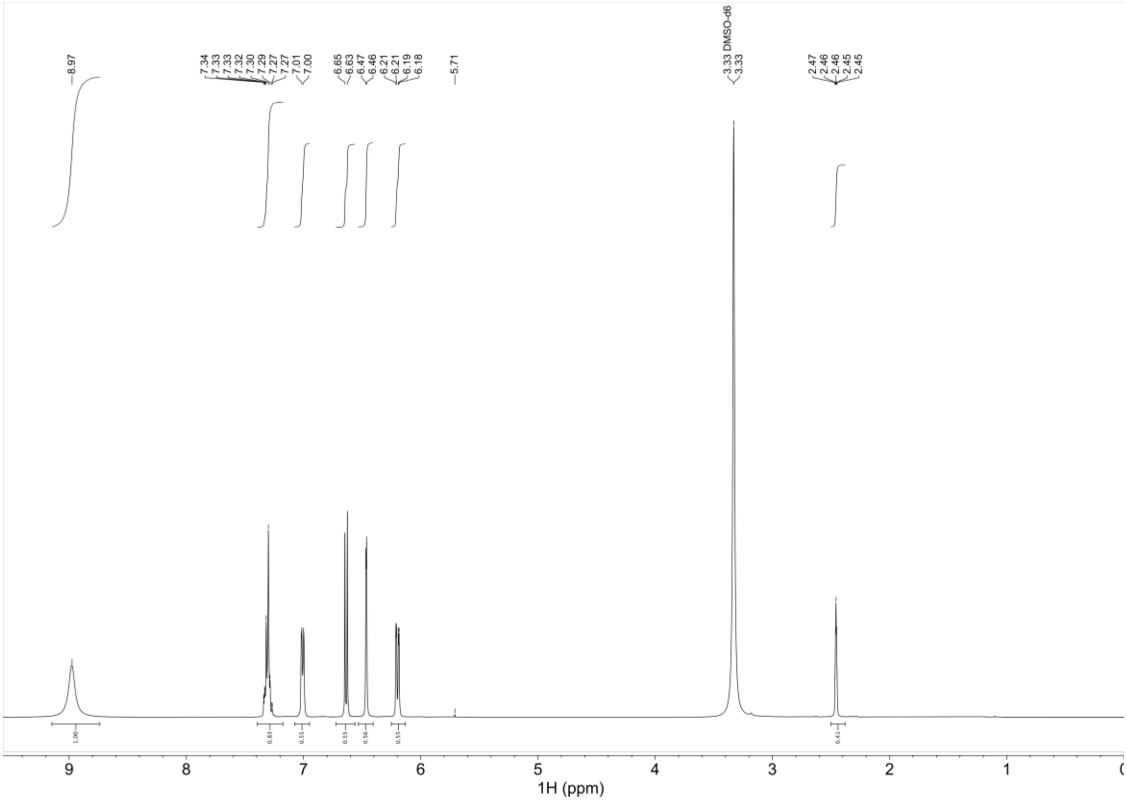

Figure S2. ^{1}H-NMR of TPBB, DMSO-d_6

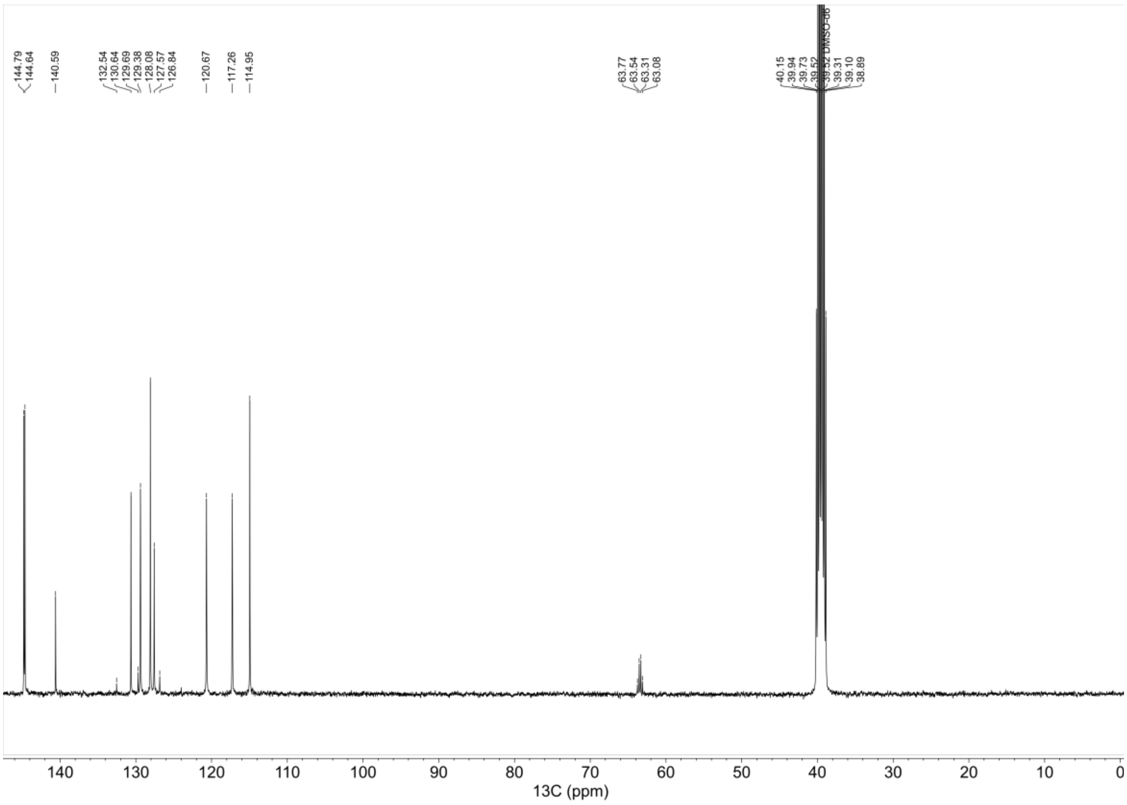

Figure S3. ^{13}C-NMR of TPBB, DMSO-d_6

1.2. PIMs synthesis

1.2.1. PIM-1

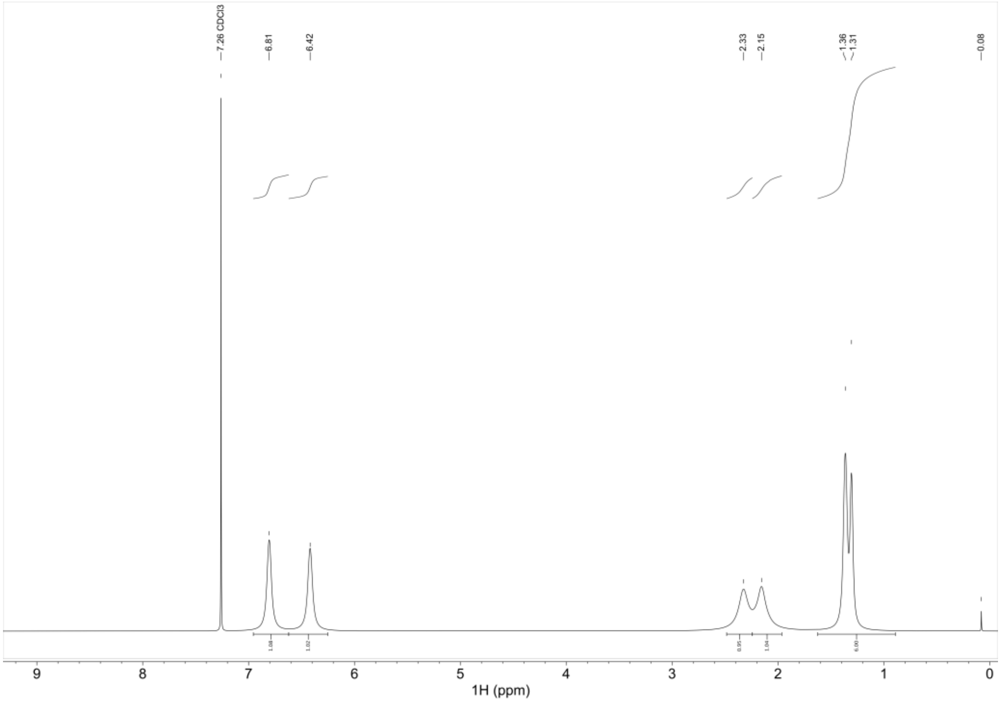

Figure S4. ^{1}H-NMR of PIM-1, $CDCl_3$

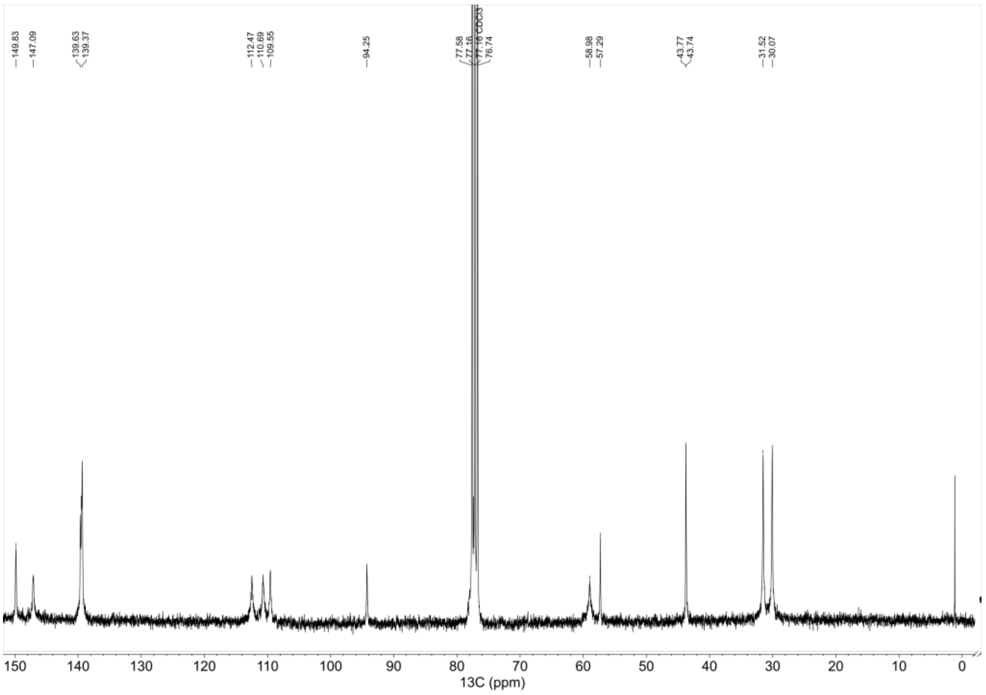

Figure S5. ^{13}C-NMR of PIM-1, $CDCl_3$

1.2.2. PIM-Ki. This synthesis has followed a similar protocol to PIM-1. 4,4'-(2,2,2-trifluoro-1-phenylethane-1,1-diyl) bis(benzene-1,2-diol) (2.01 g, 5.3 mmol), TFTPN (1.06 g, 5.3 mmol) and 8.5 mL of anhydrous dimethylacetamide was added to a three-necked 50 mL flask equipped with magnetic stirrer, Dean Stark and nitrogen inlet. The mixture was stirred for 10 minutes until all the reactants were dissolved. Once the solution was homogeneous, the flask was introduced into a 60 ºC preheated silicon bath and the temperature was increased until 90 ºC. Then, anhydrous potassium carbonate (2.21 g, 15.9 mmol) was added, and a subsequent formation of bubbles and a change from white to bright yellow color was observed. The solution was heated to 130 ºC and anhydrous toluene (7 mL) was added. Due to the increasing viscosity of the solution it was necessary to add 10 mL of anhydrous toluene to enhance the stirring and solve the non-reacted salts. After 4 hours the reaction was stopped, and then all the steps previously explained in the synthesis of PIM-1 were followed. The resulting polymer was heated at 100ºC overnight and characterized by ^{1}H and ^{13}C-NMR (Figures S6 and S7). Yield: 87%.

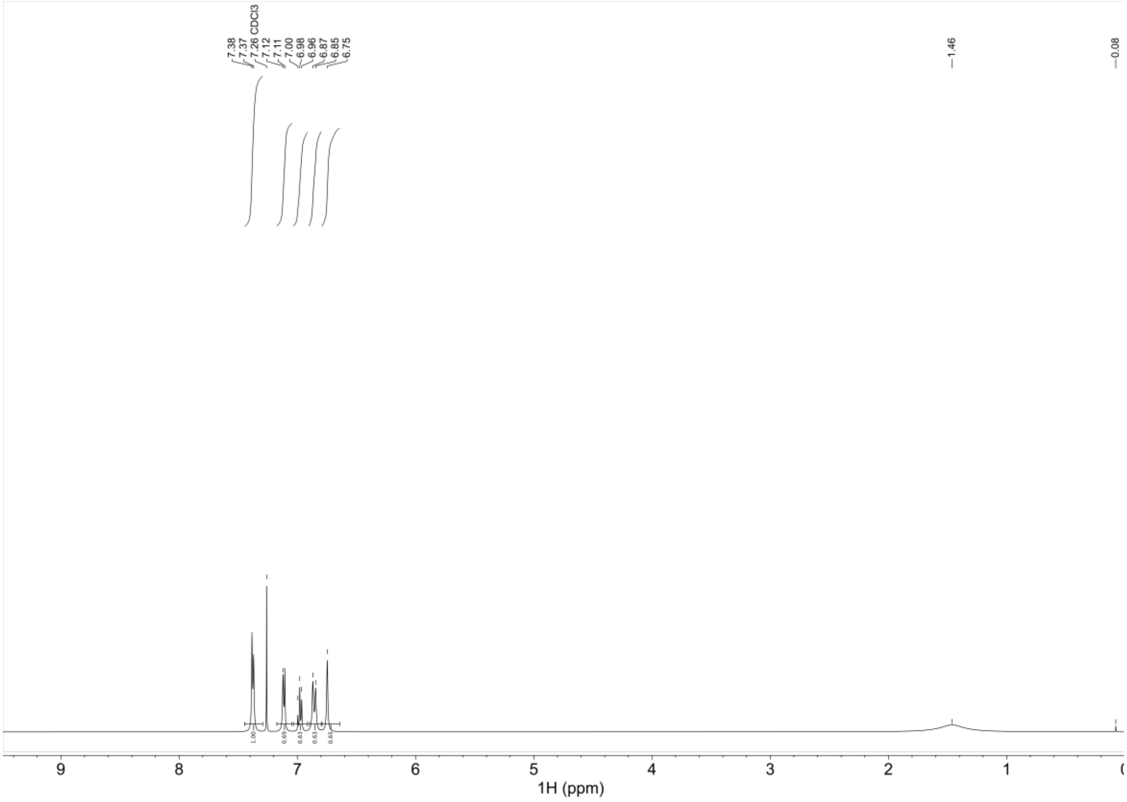

Figure S6. ^{1}H-NMR of PIM-Ki, $CDCl_3$

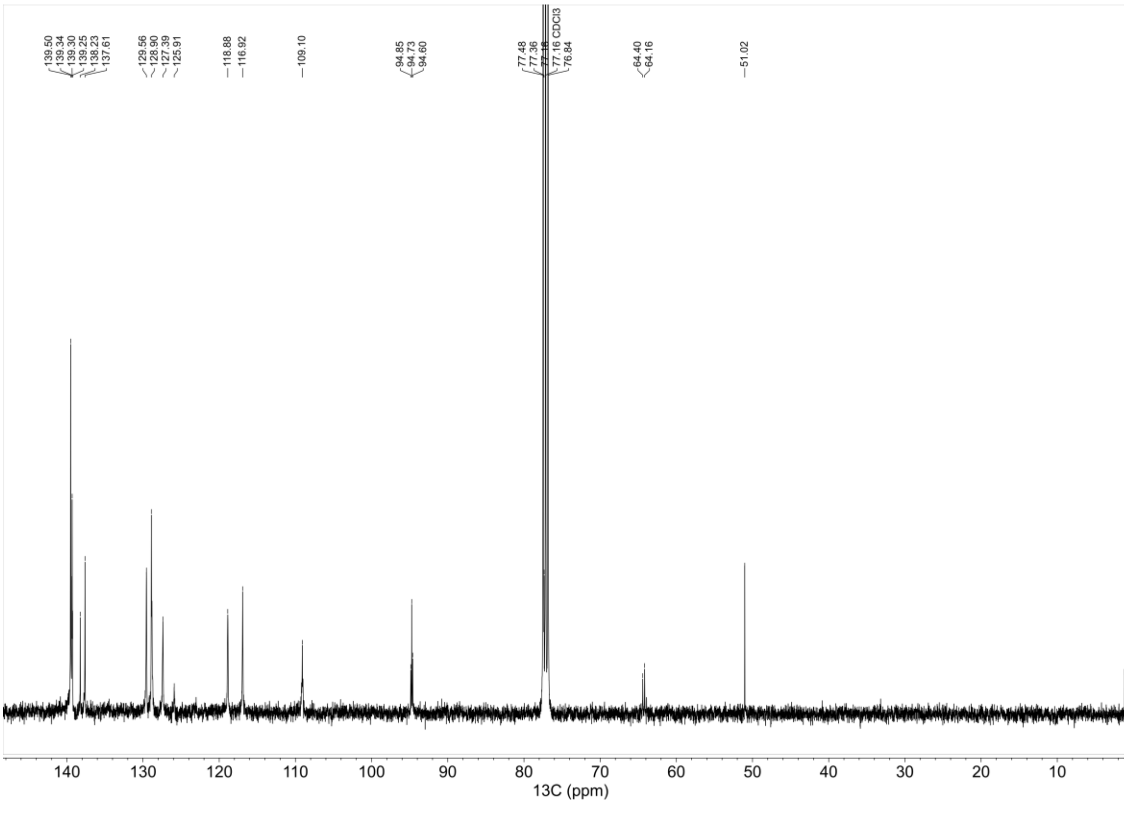

Figure S7. ^{13}C-NMR of PIM-Ki, $CDCl_3$

1.2.3. PIM-KiPY. 4,4'-(2,2,2-trifluoro-1-phenylethane-1,1-diyl)bis(benzene-1,2-diol) (1.93 g, 5.1 mmol), 2,3,5,6-tetrafluoroisonicotinonitrile (0.91 g, 5.1 mmol) and 8.5 mL of anhydrous dimethylacetamide was added to a three-necked 50 mL flask equipped with a magnetic stirrer, a Dean Stark apparatus and a nitrogen inlet. The mixture was stirred for 10 minutes until all the reactants were dissolved. Once the solution was homogeneous, the flask was introduced into a 60 ºC preheated silicon bath and the temperature was increased until 90 ºC. At this moment, anhydrous potassium carbonate (2.14 g, 15.4 mmol) was added and the formation of bubbles and a change to yellow was observed. The solution was heated to 130 ºC and anhydrous toluene (7 mL) was added. During the reaction, some additions of anhydrous toluene were needed. After 3 hours the reaction was finished and, after all the previously explained purification steps, PIM-KiPY polymer was obtained in good yield. The resulting polymer was heated at 100 ºC overnight and characterized by ^{1}H and ^{13}C-NMR (Figures S8 and S9). Yield: 79%.

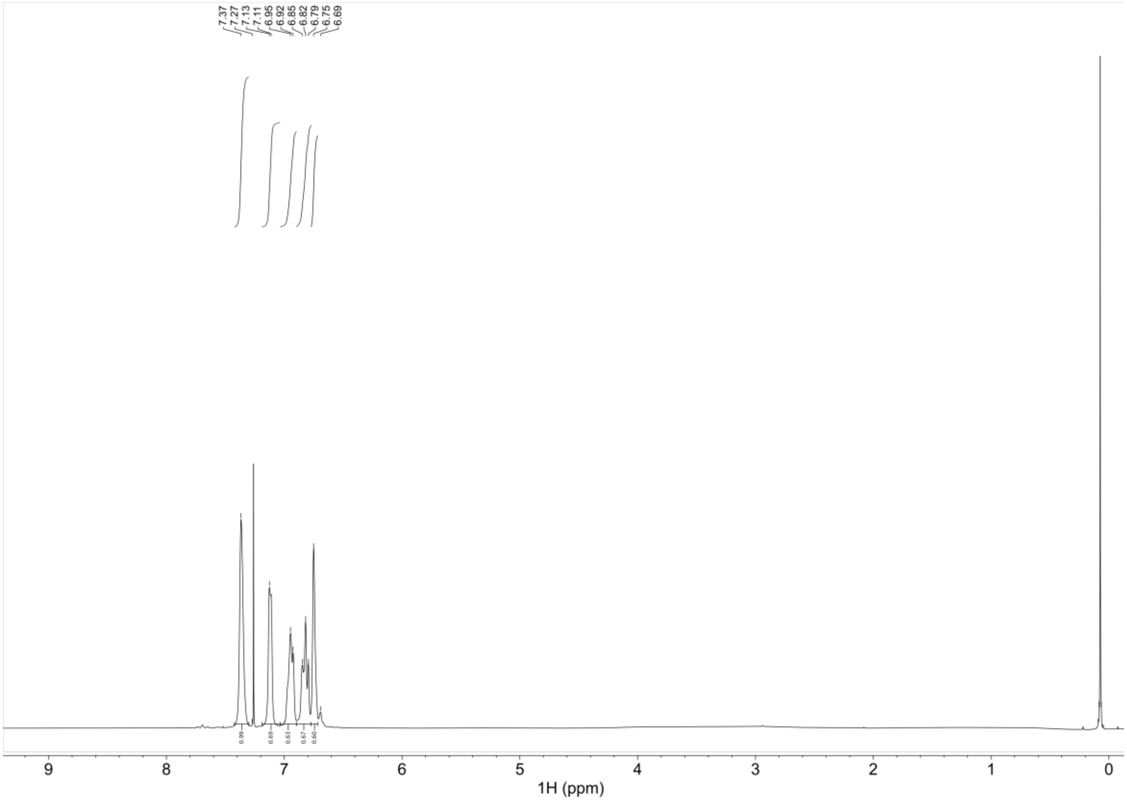

Figure S8. ^{1}H-NMR of PIM-KiPY, $CDCl_3$

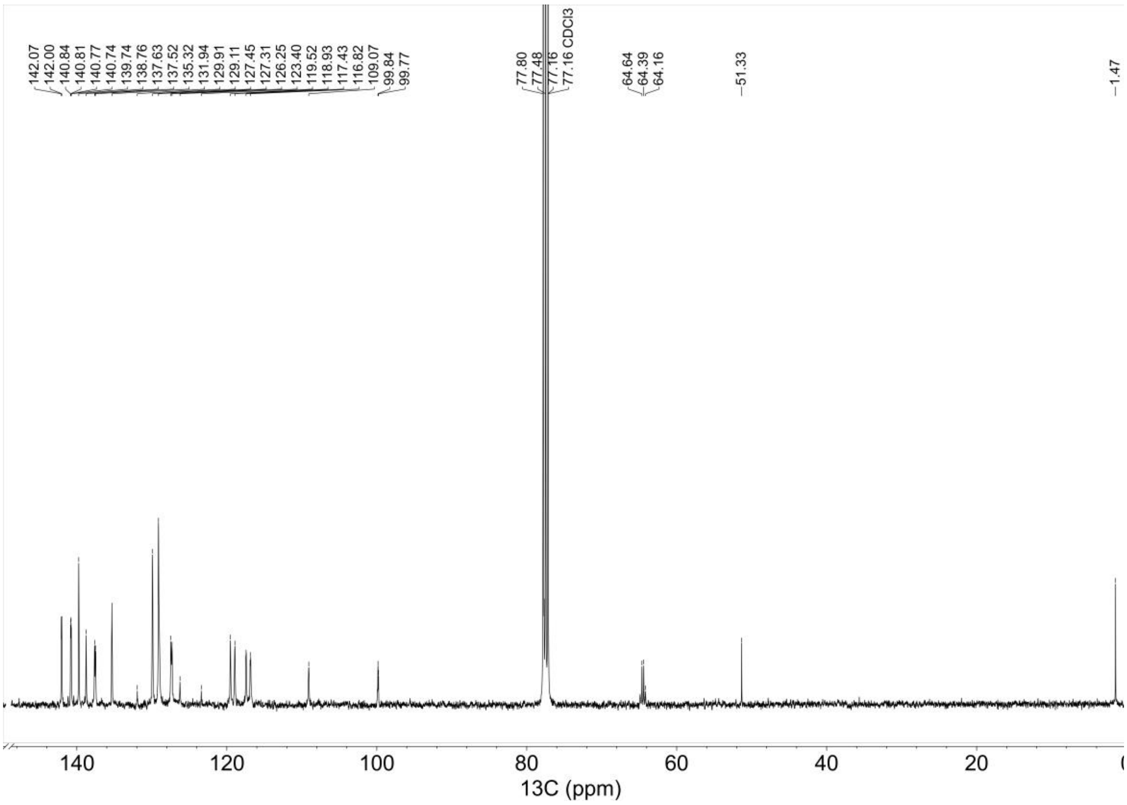

Figure S9. ^{13}C-NMR of PIM-KiPY, $CDCl_3$

1.2.4. PIM1-PIMKi-CO-11 copolymer. A copolymer containing TTSBI and TPBB in the same ratio has been synthesized. 4,4'-(2,2,2-trifluoro-1-phenylethane-1,1-diyl)-bis(benzene-1,2-diol) (0.33 g, 0.87 mmol), TFTPN (0.35 g, 1.7 mmol), TTSBI (0.29 g, 0.87 mmol) and 8.5 mL of anhydrous dimethylacetamide was added to a three necked 50 mL flask equipped with magnetic stirrer, Dean Stark and nitrogen inlet. The mixture was stirred for 10 minutes until all the reactants were dissolved. Once the solution was homogeneous, the flask was introduced into a 60ºC preheated silicon bath and the temperature was increased to 90 ºC. At this moment, anhydrous potassium carbonate (0.73 g, 5.2 mmol) was added and the formation of bubbles and a change to yellow was observed. The solution was heated to 130 ºC and anhydrous toluene was added. During the reaction, some additions of anhydrous toluene were needed. After 3 hours the reaction was finished and after all the purification steps a yellow powder was obtained. The resulting polymer was heated at 100 ºC overnight and characterized by ^{1}H and ^{13}C-NMR (Figures S10 and S11). Yield: 83%.

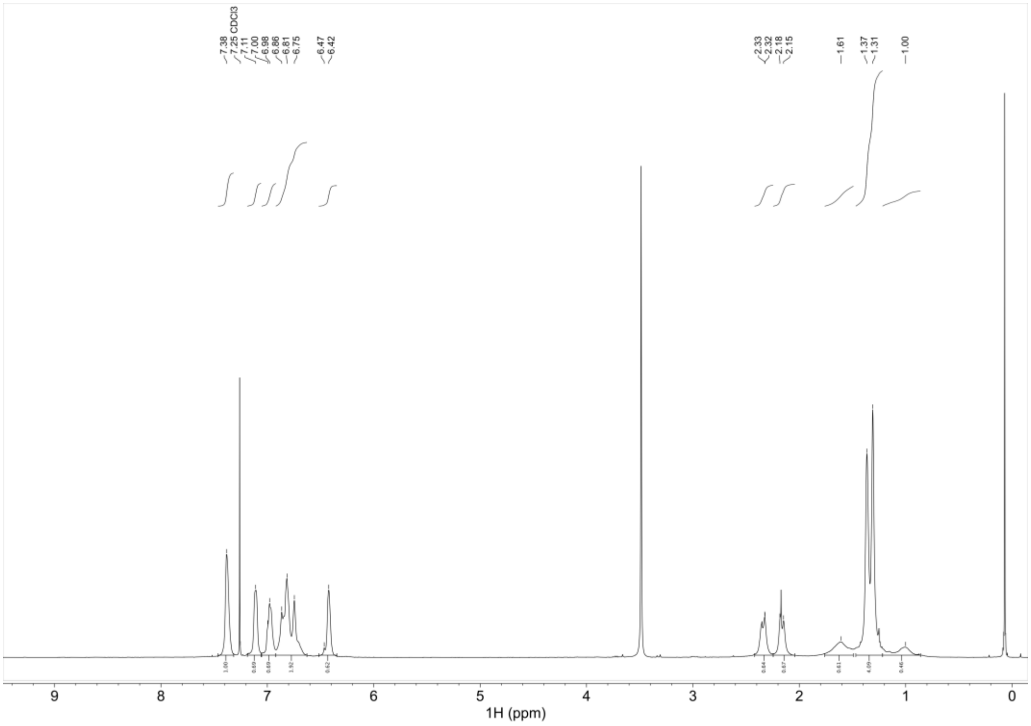

Figure S10. ^{1}H-NMR of PIM1-PIMKi-CO-11, $CDCl_3$

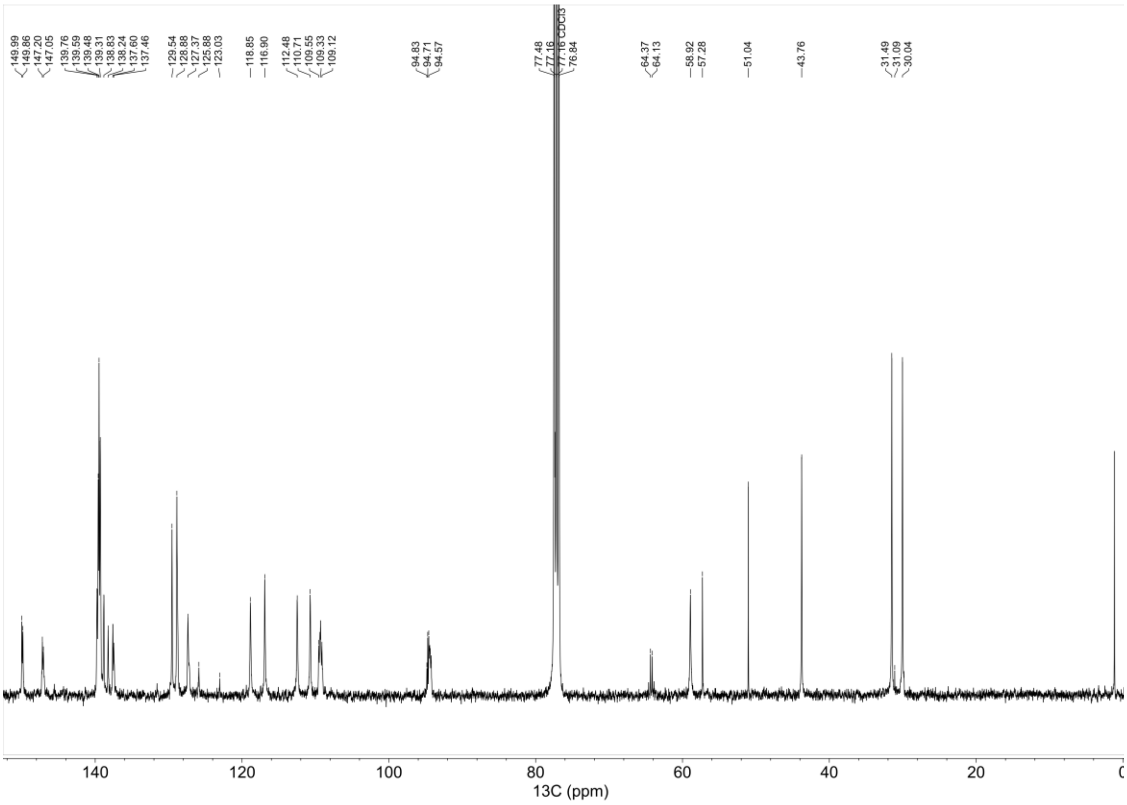

Figure S11. ^{13}C-NMR of PIM1-PIMKi-CO-11, $CDCl_3$

1.2.5. PIM-Ki-COOH

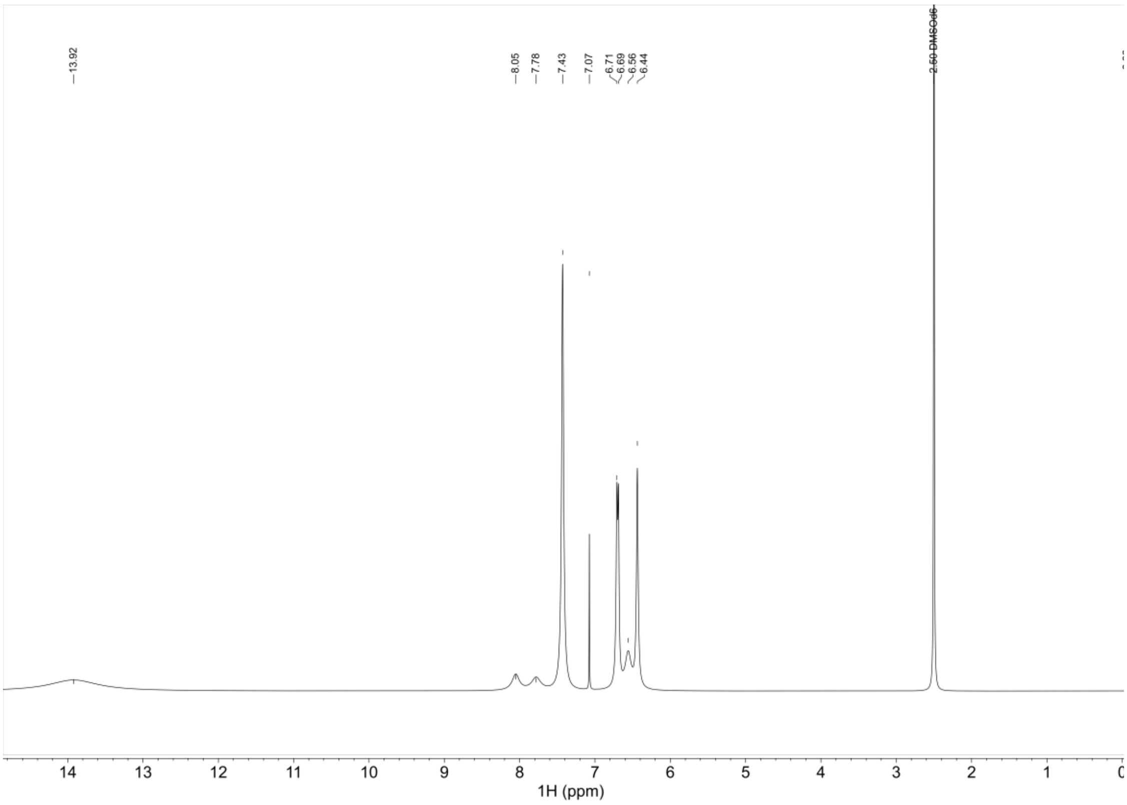

Figure S12. ^{1}H-PIM-Ki-COOH, DMSO-d_6

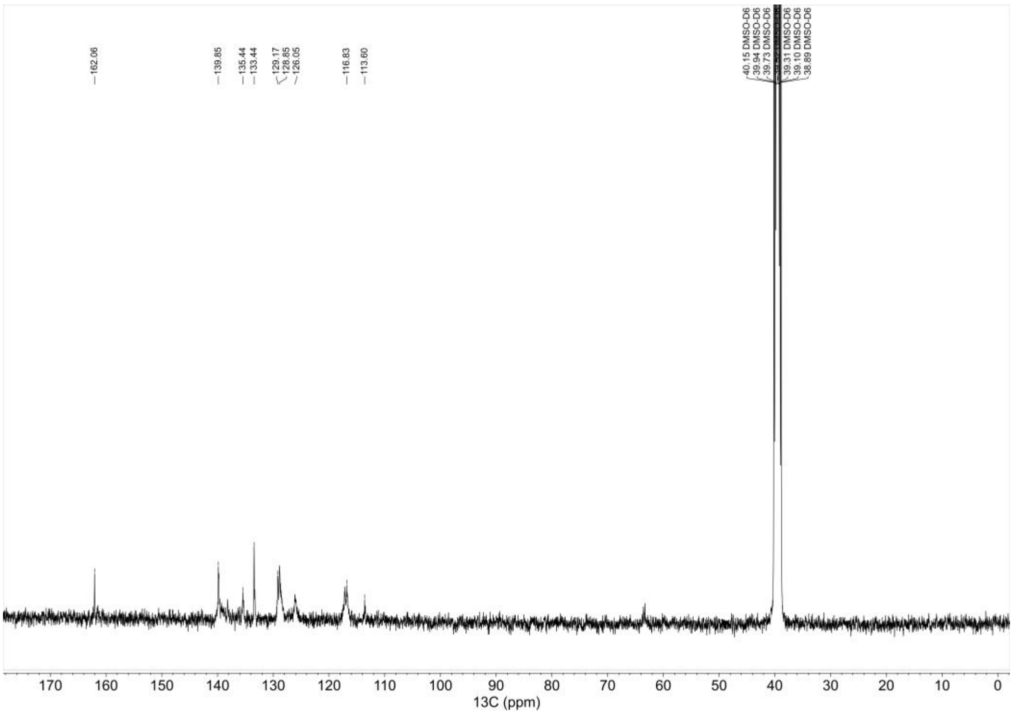

Figure S13. ^{13}C-PIM-Ki-COOH, DMSO-d_6

1.3. Film casting: solvent solubility of PIM-1, PIM-Ki, PIM-KiPY, PIM1-PIMKi-CO-11 and PIM-Ki-COOH. 2 mg of each polymer were dissolved in different polarity solvents and stirred at room temperature overnight. PIM-1, PIM-Ki, PIM-KiPY, PIM1-PIMKi-CO-11 are soluble in slightly and moderate polar solvents such as $CHCl_3$, DCM and THF. However, PIM-Ki-COOH is a higher polar polymer, so it is soluble in moderate and highly polar solvents such as THF, DMF or DMSO.

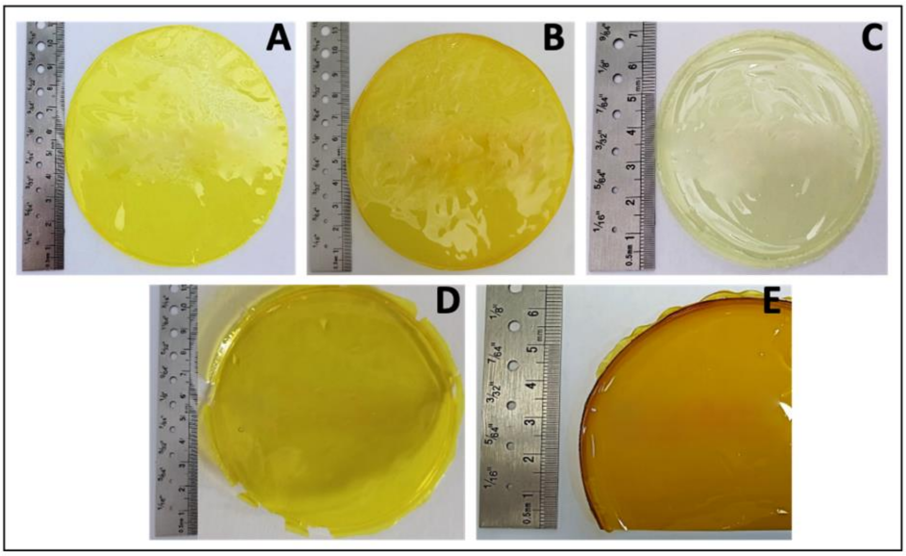

Figure S14. Polymer films casted in THF. (A) PIM-1; (B) PIM-Ki; (C) PIM-KiPY; (D) PIM1-PIMKi-CO-11; (E) PIM-Ki-COOH

1.4. Chemical and physical characterization

1.4.1. ATR-FTIR of PIMs

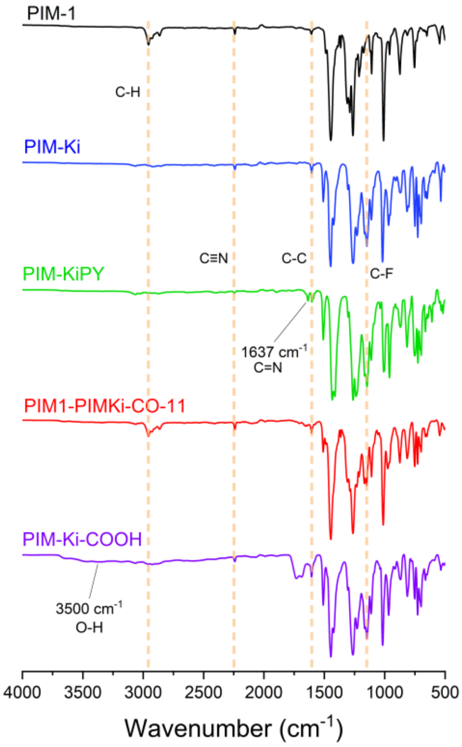

Figure S15. ATR-FTIR spectra of PIMs and carboxylated PIM films. All the spectra were normalized to 1446 cm^{-1} peak and shifted for better comparison.

1.4.2. Thermogravimetric analysis

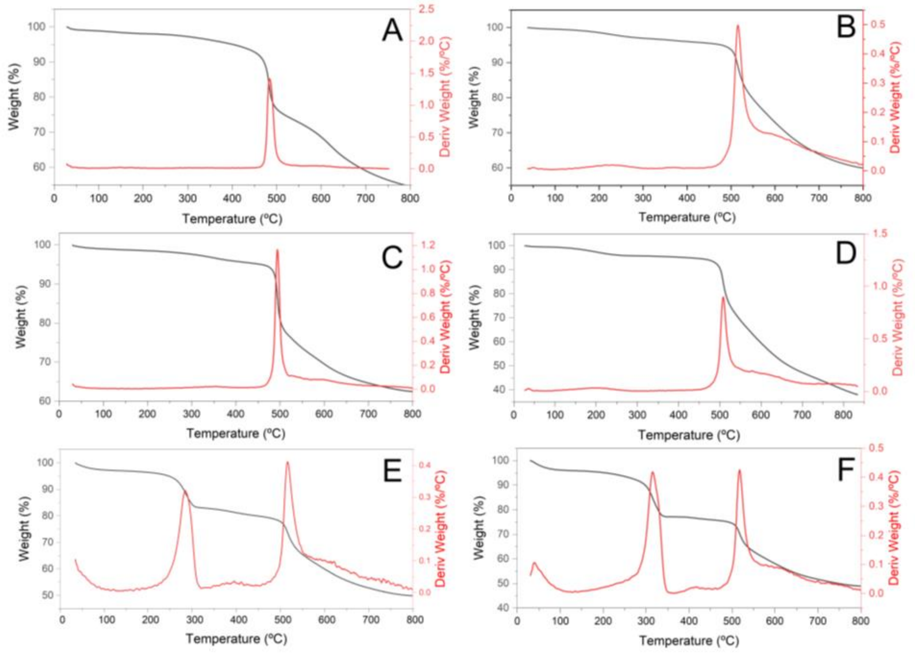

Figure S16. Thermogravimetric analysis curves of PIM-1 (A), PIM-Ki (B), PIM-KiPY (C), PIM1-PIMKi-CO-11 copolymer (D) and PIM-Ki-COOH (65-100%) (E and F respectively). All the polymers have been dried at 120ºC before doing the analysis.

1.4.3. Mechanical tests

Uniaxial tensile tests were carried out in an MTS Synergie-200 testing instrument equipped with a 100 N load cell at room temperature. Rectangular test pieces (5 mm width and 30 mm length) of 40 µm thickness films were cut and each sample was subjected to a tensile load applied at a rate of 5 mm min^{-1} until fracture (Table S1).

PIM-Ki and PIM-KiPY membranes show very similar Young's modulus, 2.0 ± 0.3 and 1.9 ± 0.5 (GPa) respectively, which are higher than the reported ones for the benchmark polymer, PIM-1. PIM-Ki-COOH (65%) shows a higher Young's modulus not only due to its capacity to form H bonds (which makes it more resistant), but also because it conserves the rigidity. This did not happen with the 100% modified PIM-Ki-COOH, since the presence of carboxylic groups seems to result in a lower mechanical modulus, and thus a lower mechanical resistance.

Sample	Stress at break (MPa)	Strain at break (%)	Young's modulus (GPa)
**PIM-1**	76 ± 5	6.6 ± 2.6	2.0 ± 0.2
**PIM-Ki**	83 ± 14	7.1 ± 1.9	2.0 ± 0.3
**PIM-KiPY****	104 ± 16	11.9 ± 1.9	1.9 ± 0.5
**PIM1-PIMKi-CO-11**	19 ± 1	2.0 ± 0.1	1.2 ± 0.2
**PIM-Ki-COOH (65%)**	45 ± 1	8.2 ± 0.7	2.2 ± 0.3
**PIM-Ki-COOH (100%)**	66 ± 9	7.1 ± 1.9	1.6 ± 0.1

Table S1. Stress and strain at break and Young's Modulus from PIMs.

1.4.4. Gas permeation

Pure gas permeation measurements were carried out on PIMs membranes. Permeability (P) was measured for different gases (N_2, O_2, CO_2, He and CH_4). Permeation flow was measured using a custom-made constant volume/variable pressure apparatus at 25ºC and 1 bar.[3] Before the measurements the membranes were placed in the system and maintained under high vacuum overnight to remove traces of humidity or other species. The permeate pressure was recorded as a function of time until a steady state was reached. The permeability coefficient, P, was calculated as follows (eq. S1):

$$P = \frac{273Vl}{76ATp_o} \cdot \left[\left(\frac{dp(t)}{dt}\right)_{ss} - \left(\frac{dp(t)}{dt}\right)_{leak}\right] \qquad \text{(S1)}$$

Where V is the downstream volume (cm^3), T is the temperature (K), l is the membrane thickness (cm), A is the effective area (cm^2) of the membrane, p_o is the upstream pressure (bar), $(dp(t)/dt)_{ss}$ is the steady state rate of the permeate pressure (mbar s^{-1}), and $(dp(t)/dt)_{leak}$ is the system leak rate (mbar·s^{-1}), which is less than 1% of $(dp(t)/dt)_{ss}$. The standard conditions used were 76 cmHg and 273.15 K for pressure and temperature.

Selectivity was also calculated as the ratio between two gas permeabilities ($\alpha_{A,B}$), eq S2

$$\alpha_{A,B} = \frac{P_A}{P_B} \qquad \text{(S2)}$$

The gas permeability of membranes is very sensitive to film preparation conditions and pre-treatment, therefore all the films were prepared and measured in the same conditions. Since PIM-Kis swell methanol, they were not introduced in this alcohol before the measurements; instead, they were heated in a temperature ramp from 30 to 120 ºC in 12 hours. Gas permeabilities and selectivities of PIMs are shown in Table S2. They follow a clear relationship between permeability and selectivity, one of them is gained at the cost of a loss in the other one, and vice versa. The values obtained are close to some of other reported PIMs measured in the same conditions (aged state).

**Sample**	**Permeability (P_i, Barrer)**			**Selectivity ($\alpha_{i/j}=P_i/P_j$)**
	He	CO_2	CH_4	$\alpha_{CO2/CH4}$
**PIM-1**	1108	2815	169	16.7
**PIM-1**[4]	-	2300	125	18.4
**PIM-PI-9**[5]	-	2180	170	12.8
**PIM-1-COOH**[6]	-	620	-	-
**PIM-Ki**	307	770	31	24.8
**PIM-KiPY**	211	511	18	28.3

Table S2. Gas permeability and selectivity of PIM-1, PIM-Ki and PIM-KiPY at 30 ºC and 1 bar. The membranes were subject a thermal treatment heating gradually up to 120 ºC and maintained at 120 ºC for 12 h.

Section 2. Ion transport through PIM membranes

2.1. Set up

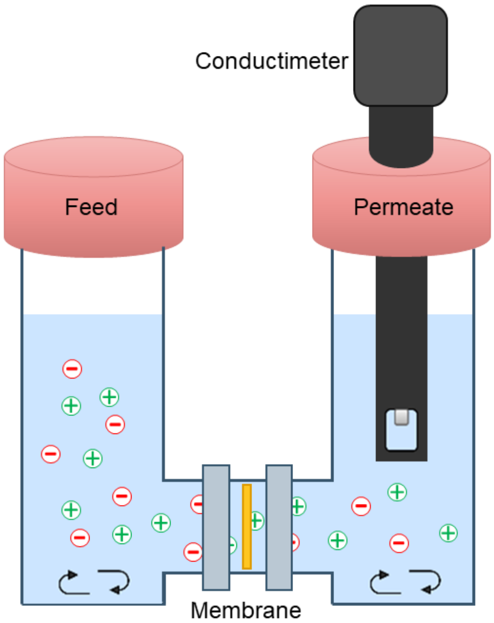

Figure S17. Experimental H-cell set-up for ion diffusion tests across the PIM membrane with the conductivity-meter probe located in the permeated compartment.

The conductivity value in the permeate tank was continuously measured every hour by using a conductivity-meter (Eutech™ PC 2700, Thermo Scientific).

The ionic conductivity of the salt solution in the permeate tank increased due to the time dependent concentration gradient between the two tanks, which is the driving force promoting the ion transport across the membrane. A linear relationship between the salt concentration and the conductivity value of the different aqueous solutions was evidenced, allowing the interpolation in the calibration curves and the quantification of the ion concentration in the permeate side of the membrane.

2.2. Ion diffusion through a non-modified PIM-Ki membrane experiment.

Since PIM-Ki structure does not includes any hydrophilic functional groups, conductivity does not increase with time, which means that water cannot pass through the membrane. This proves that modifying the membranes with hydrophilic groups is a must if we want to use them as permeate membranes.

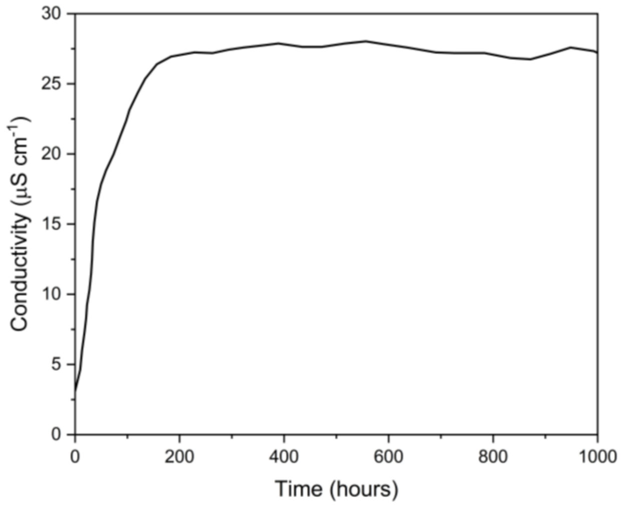

Figure S18. LiCl ion transport curve using a non-modified membrane of PIM-Ki. As it can be seen, conductivity only increases 20 μS, which is not significant, so it is not an ion conducting membrane in aqueous media.

2.3. Conductivity concentration calibration plots. To correlate the conductivity measured with the salt concentration in the permeate solution over time, a calibration curve was established. For that, the conductivity of several salt aqueous solutions ranging from 0 M to 5.5 M were measured using the conductivity-meter (Eutech™ PC 2700, Thermo Scientific). Figure S19 shows the conductivity-concentration plots for solutions of LiCl, NaCl and KCl.

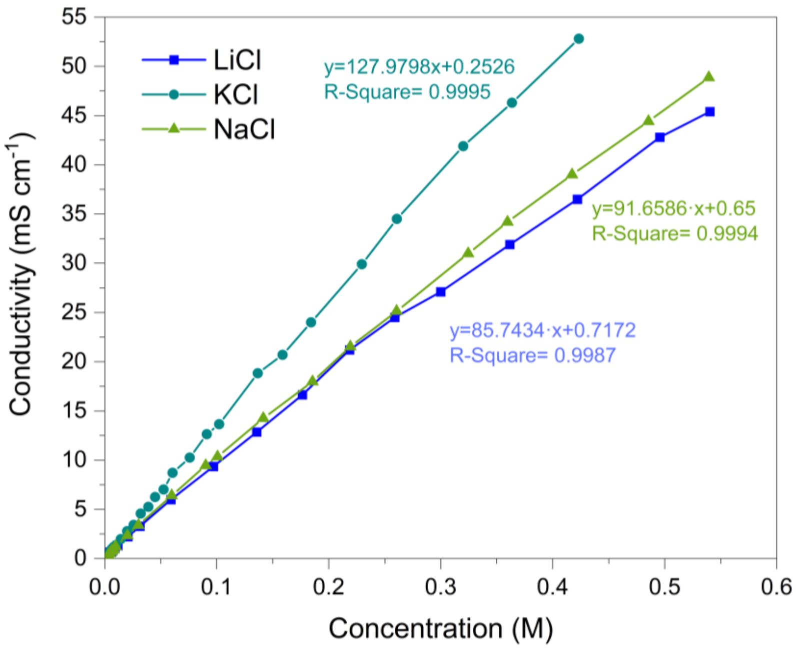

Figure S19. Conductivity-concentration calibration plots of the saline aqueous solutions of LiCl, NaCl, and KCl.

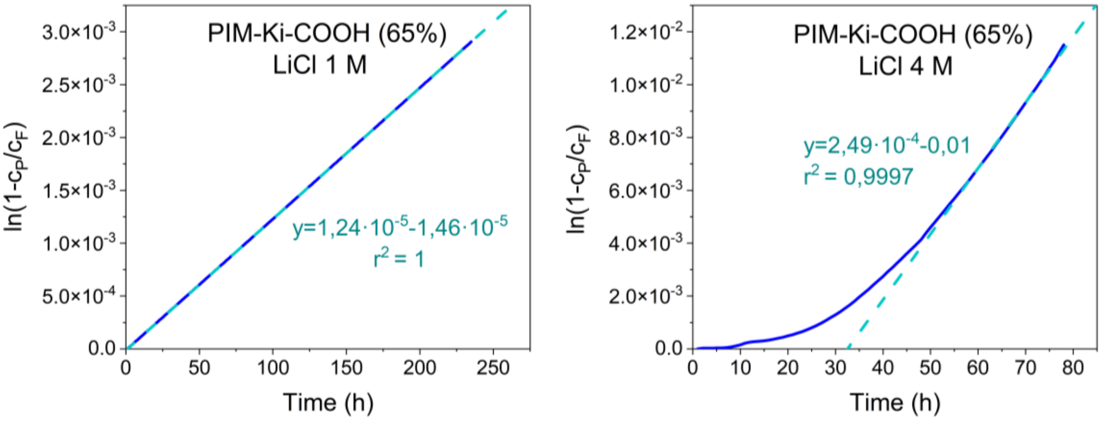

Figure S20. Fitting of the permeation curve used to calculate the permeability in LiCl 1-4M tests using PIM-Ki-COOH (65%) membrane.

2.4. Electrochemical Impedance Spectroscopy (EIS) experiments.

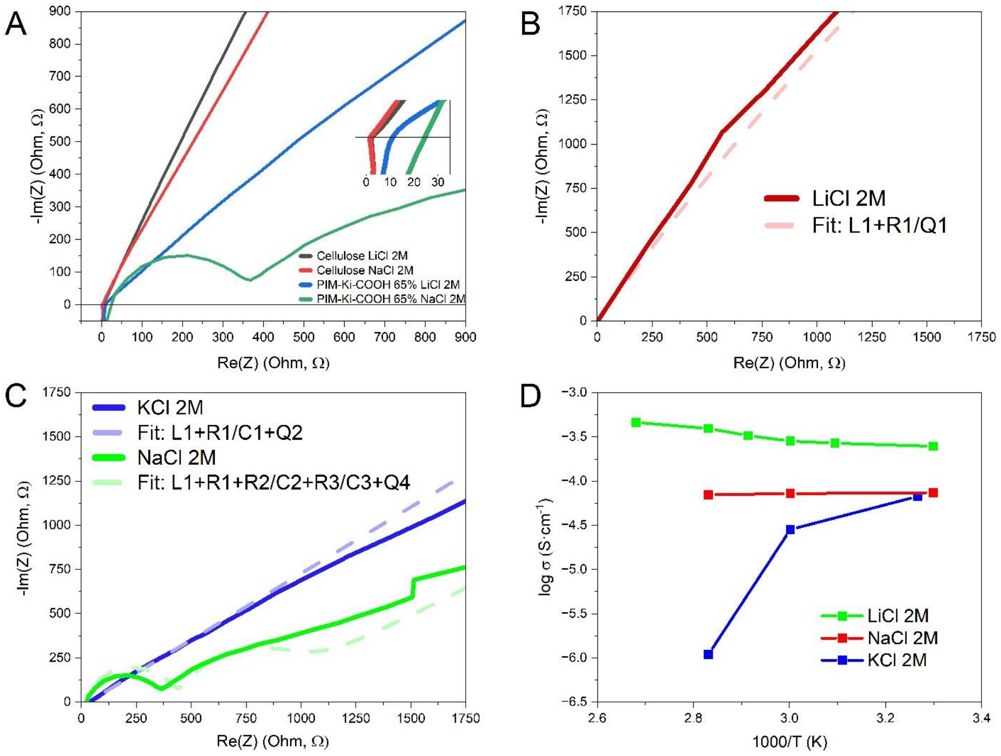

Figure S21. (A) EIS Nyquist plots using cellulose and PIM-Ki-COOH (65%) membrane and LiCl or NaCl 2M electrolytes; (B) Fitting of the Nyquist plot using a PIM-Ki-COOH (65%) membrane and LiCl 2M electrolyte; (C) Fitting of the Nyquist plots using a PIM-Ki-COOH (65%) membrane and KCl 2M and NaCl 2M electrolytes; (D) Representation of the conductivities using different electrolytes and PIM-Ki-COOH (65%) membrane.

Section 3. Inline NMR diffusion and crossover experiments

3.1. Experimental set up.

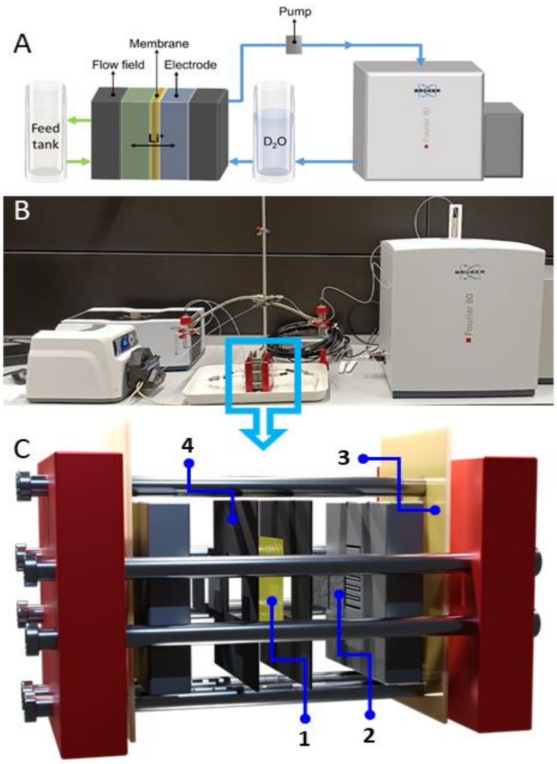

Figure S22. (A) Inline $^{7}Li/^{35}Cl$-NMR diffusion and ^{1}H-NMR crossover experiments set up using a Bruker Fourier 80MHz NMR magnet; (B) Picture of the actual set up. It is possible to see the peristaltic pumps (left), the cell (center) and the NMR magnet (right); (C) Scribner® flow cell breakdown: 1, PIM-Ki-COOH (65%) membrane; 2, carbon paper; 3, current collector; 4, rubber gasket.

3.2. Ion exchange process to convert $K_{3/4}Fe(CN)_6$ to $Li_{3/4}Fe(CN)_6$

$Li_3Fe(CN)_6$ and $Li_4Fe(CN)_6$ were prepared as described in previous reports[7].

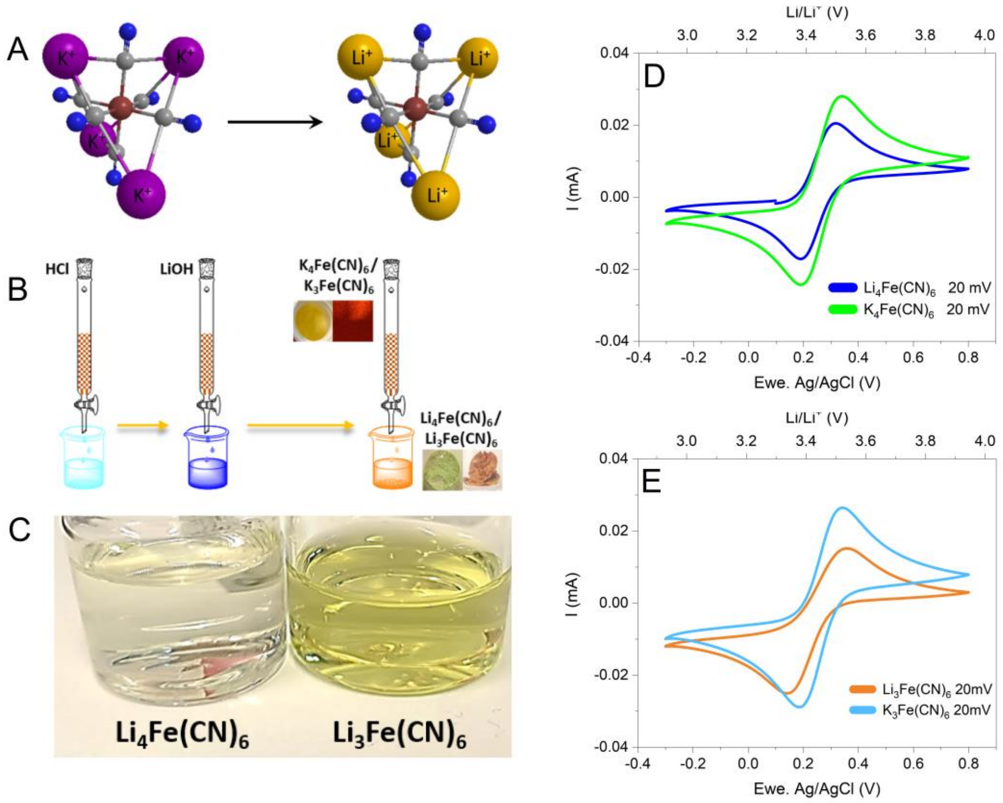

Figure S23. (A) Ion exchange scheme from $K_{3/4}Fe(CN)_6$ to $Li_{3/4}Fe(CN)_6$; (B) ion exchange procedure; (C) differences in solution color between the two compounds after the ion exchange process; (D) and (E), cyclic voltammetry of the aqueous solutions containing the redox species before and after the ion exchange[7].

3.2.1 Elemental analysis of lithium salts after ion exchange process was carried out to confirm the presence of lithium in the compounds and therefore, to conclude that the ion exchange process has been successfully performed. Experimental $Li_3Fe(CN)_6$ lithium content, 60.1 ± 0.8 $mg \cdot g^{-1}$ and theoretical, 90 $mg \cdot g^{-1}$; Experimental $Li_4Fe(CN)_6$ lithium content, 80.9 ± 0.8 $mg \cdot g^{-1}$ and theoretical, 110 $mg \cdot g^{-1}$.

3.3. Chemical shift variation in the presence of ionic species

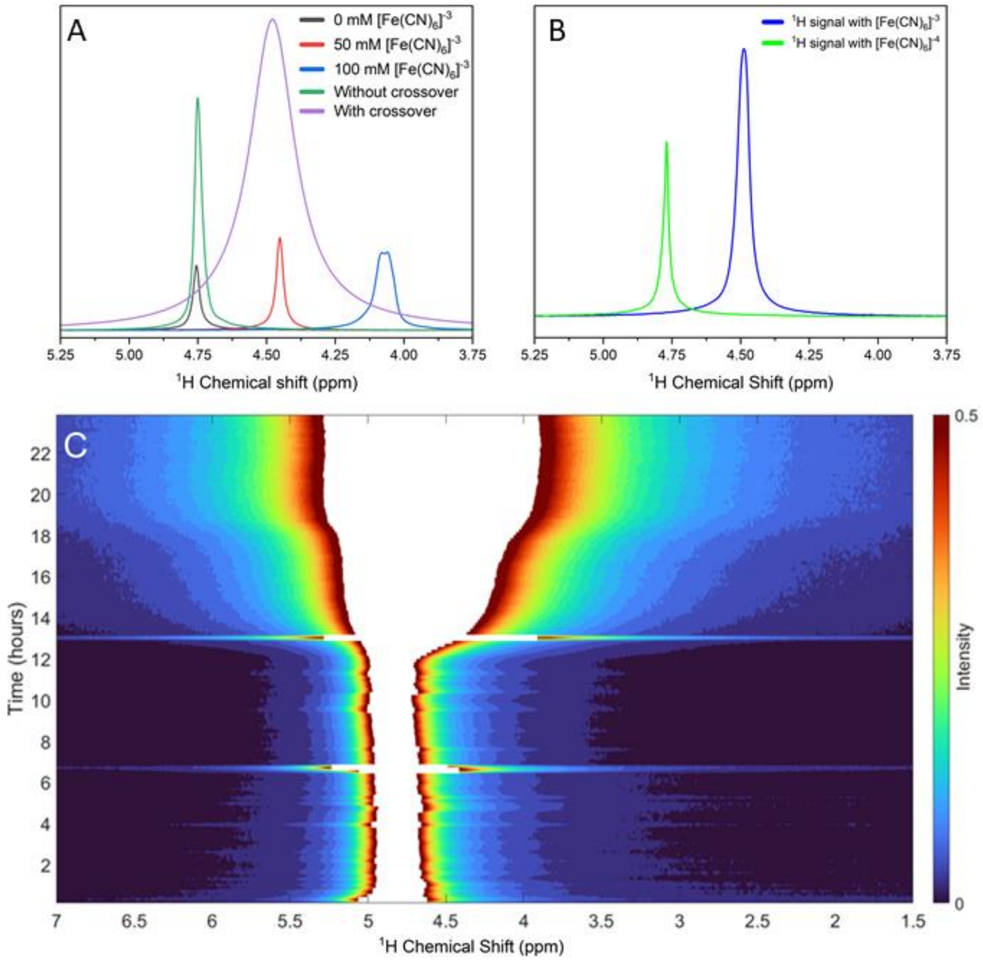

Figure S24. (A) ^{1}H-NMR spectra of H_2O protons at different $[Fe(CN)_6]^{-3}$ concentrations and signals from the experiments with (purple) and without $[Fe(CN)_6]^{-3}$ crossover through the membrane (green); (B) ^{1}H-NMR spectra of H_2O protons in the presence of $[Fe(CN)]^{-3}$ and $[Fe(CN)]^{-4}$. The paramagnetic properties of $[Fe(CN)_6]^{-3}$ cause a displacement of the chemical shifts of the solvent, inducing a difference on the bulk magnetization.; (C) in this ^{1}H-NMR spectrum of an experiment with $[Fe(CN)_6]^{-3}$ crossover is possible to see what happens when the membrane is not selective. The signal starts to get broader and to shift after 12 hours, once the redox $[Fe(CN)_6]^{-3}$ specie starts to pass through it. (A) and (B) spectra were recorded using an 80 MHz (Bruker) spectrometer; and (C) was obtained with a 43 MHz (Magritek) spectrometer.

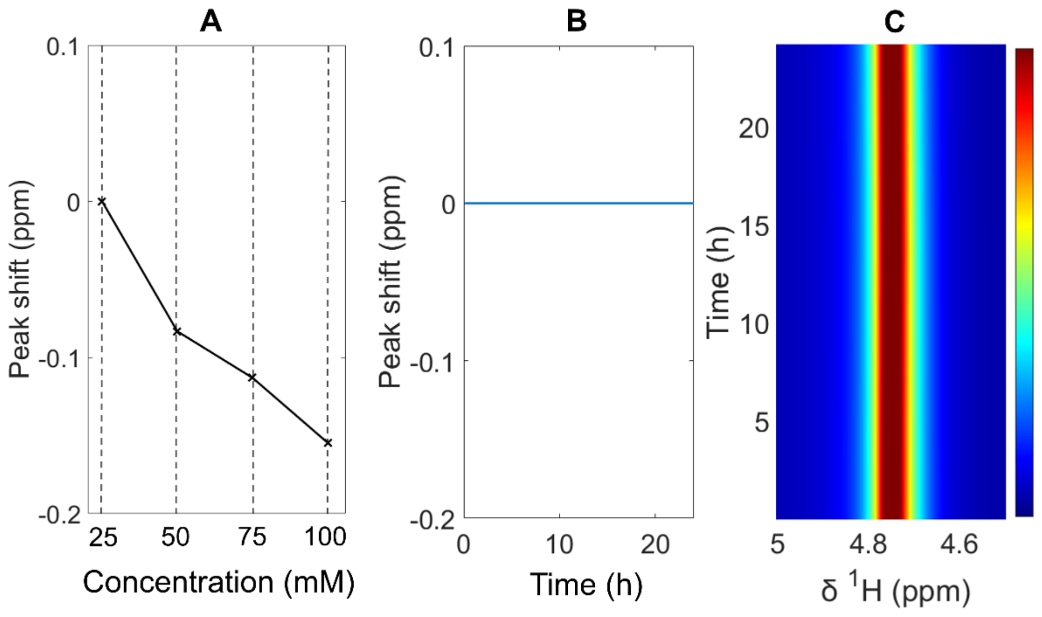

Figure S25. Comparison between the chemical shifts of static and inline ^{1}H-NMR spectra of $Li_3Fe(CN)_6$ done in an 80 MHz (Bruker) spectrometer. (A) Variation of chemical shift versus salt concentration, leading to decreasing value for the chemical shift at more concentrated solutions. (B) Variation of the chemical shift for the inline ^{1}H-NMR experiment, showing no apparent drifting of the solvent. (C) zoomed in contour plot of the stacked ^{1}H-NMR spectra for the inline experiment, which shows the stability of the proton resonance for the solvent. As mentioned in the main text, the idea for this experiment is to see the changes in bulk magnetization due to the presence of paramagnetic species such as iron (III). This is interesting for us since the drift of the solvent peak can be used to subtract the concentration of the crossing paramagnetic ions by applying the Evan's method[8]. Previous publications show the developed formula, which relates directly the chemical shift with the magnetic susceptibility[9,10]. The relationship between the changes of the chemical shift and the concentration are proportional.

3.4. Calibration of inline experiments

Pseudo-2D measurements were conducted to track temporal changes in the feeding tank. Acquisition commenced upon complete filling of the flow cell with the electrolyte solution. Spectral acquisition was achieved by conducting simple pulse-acquire experiments with a pulse angle of 90º. Prior to experiments, all electrolyte solutions underwent calibration to determine relevant NMR parameters and ensure sufficient resolution. In the case of Li^+ transport study, a recycle delay (d1) of 5 seconds, an acquisition time (aq) of 5 seconds, and 8 scans (ns) were chosen. The pulse sequence yielded a ^{7}Li-NMR spectrum every 80 seconds ($(d1 + aq) * ns = 80s$). Subtracting the Li^+ concentration transported across the membrane was possible by calibrating the signal intensity. Calibration was

achieved by flowing a LiCl solution with a known concentration (0.5M) under identical flow-rate and acquisition parameters as previously described. For the anthraquinone-2,7-disulfonic acid dilithium salt and $Li_3Fe(CN)_6$ electrolyte solutions, acquisition parameters (d1=70s, aq=2s, ns=4) resulted in a ^{1}H-NMR spectrum every 6 minutes. Proton spectra were referenced to the chemical shift of residual H_2O in deuterium oxide at 4.8 ppm.

Section 4. MAS Solid State NMR

4.1. Identification of non-protonated carbons

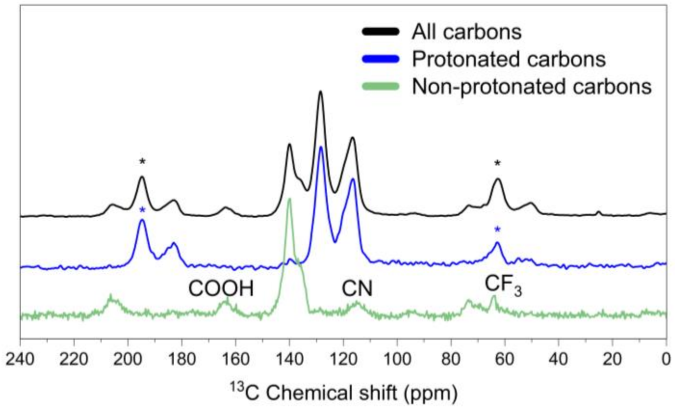

Figure S26. MAS-ssNMR allow us to identify which of the carbon signals comes from protonated carbons and which ones are from non-protonated ones, what facilitates to assign COOH, CN and CF_3 signals. A Bruker 300 MHz spectrometer was used for the experiment. *Spinning side bands

Section 5. Half-cell experiments

5.1. Galvanostatic experiment using a symmetric cell and PIM-Ki-COOH (65%) membrane

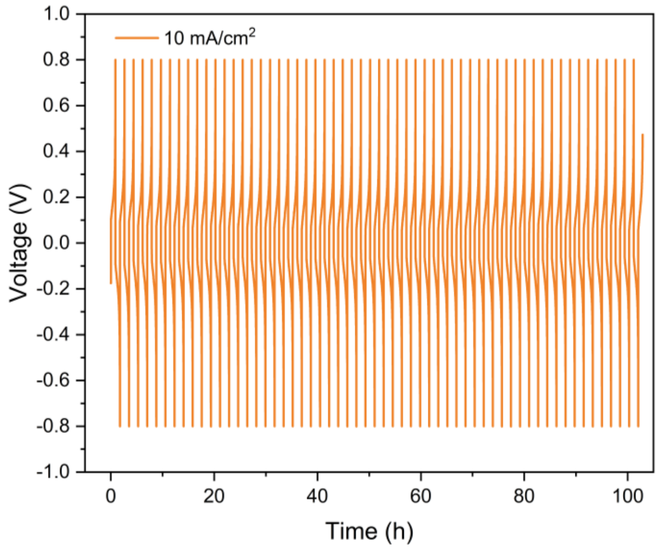

Figure S27. Charge-discharge curves at a current density of 10 mA·cm^{-2}.

Section 6. Comparative tables of permeability and electrochemical properties.

**Membrane**<br><br>**PIM-Ki-COOH**	**Salt**	**Permeability ($cm^2 \cdot s^{-1}$)/ Conductivity, 30 ºC ($S \cdot cm^{-1}$)**	**Ref.**
50h (65%)	LiCl	2.4 10^{-9}/2.5 10^{-4}	This work
	NaCl	7.38 10^{-10}/7.4 10^{-5}	This work
	KCl	1.05$10^{-9}$/6.7 10^{-5}	This work
75h (85%)	LiCl	4.80 10^{-12}	This work
150h (100%)	LiCl	4.60 10^{-12}	This work
Nafion© 117	LiCl	9.51 10^{-7}	11
Nafion© 117	NaCl	7.30 10^{-7}/20 10^{-3} (K^+)	11
SPX-BP-0.95	LiCl	8.97$10^{-7}$/ 20 10^{-3} (K^+)	11

Table S3. Comparison of the permeability and ion conductivity values of the PIM-KI-COOH membranes developed in this work with other membranes from similar research.

**Catholyte / Concentration (M)**	**Separator**	**Capacity ($A \cdot h \cdot L^{-1}$)**	**CE (%)**	**Ref.**
$Li_4Fe(CN)_6$ / 0.1	PIM-Ki-COOH (65%)	1.86	99	This work
$Li_4Fe(CN)_6$ / 2.3	Nafion 117©	61	99	7

Table S4. A comparison between the polymeric membrane developed in our work and Nafion 117© membranes implemented in a redox flow cell with $Li_4Fe(CN)_6$ as catholyte material.

References

(1) Tao, L.; Yang, H.; Liu, J.; Fan, L.; Yang, S. Synthesis and Characterization of Fluorinated Bisphenols and Tetraphenols via a Simple One-Pot Reaction. Synth Commun 2013, 43 (17), 2319–2325, DOI 10.1080/00397911.2012.705214.

(2) Tao, L.; Yang, H.; Liu, J.; Fan, L.; Yang, S. Synthesis of Fluorinated Polybenzoxazoles with Low Dielectric Constants. J Polym Sci A Polym Chem 2010, 48 (21), 4668–4680, DOI 10.1002/pola.24253.

(3) Matesanz-Niño, L.; Webb, M. T.; González-Ortega, A.; Palacio, L.; Álvarez, C.; Lozano, Á. E.; Galizia, M. Plasticization Resistant Gas Separation Membranes Derived from Polyimides Exhibiting Polyethylene-Oxide Moieties. Polymer (Guildf) 2024, 290, 126535, DOI 10.1016/j.polymer.2023.126535.

(4) Budd, P. M.; Msayib, K. J.; Tattershall, C. E.; Ghanem, B. S.; Reynolds, K. J.; Mckeown, N. B.; Fritsch, D. Gas Separation Membranes from Polymers of Intrinsic Microporosity. J Memb Sci 2005, 251, 263–269, DOI 10.1016/j.memsci.2005.01.009.

(5) Du, N.; Robertson, G. P.; Song, J.; Pinnau, I.; Guiver, M. D. High-Performance Carboxylated Polymers of Intrinsic Microporosity (PIMs) with Tunable Gas Transport Properties. Macromolecules 2009, 42 (16), 6038–6043, DOI 10.1021/MA9009017.

(6) Ghanem, B. S.; McKeown, N. B.; Budd, P. M.; Selbie, J. D.; Fritsch, D. High-Performance Membranes from Polyimides with Intrinsic Microporosity. Advanced Materials 2008, 20 (14), 2766–2771, DOI 10.1002/ADMA.200702400.

(7) Li, X.; Yao, Y.; Liu, C.; Jia, X.; Jian, J.; Guo, B.; Lu, S.; Qin, W.; Wang, Q.; Wu, X. Lithium Ferrocyanide Catholyte for High-Energy and Low-Cost Aqueous Redox Flow Batteries**. 2023, DOI 10.26434/chemrxiv-2022-hx538.

(8) Evans, D. F. 400. The Determination of the Paramagnetic Susceptibility of Substances in Solution by Nuclear Magnetic Resonance. Journal of the Chemical Society (Resumed) 1959, No. 0, 2003, DOI 10.1039/jr9590002003.

(9) Zhao, E. W.; Liu, T.; Jónsson, E.; Lee, J.; Temprano, I.; Jethwa, R. B.; Wang, A.; Smith, H.; Carretero-González, J.; Song, Q.; Grey, C. P. In Situ NMR Metrology Reveals Reaction Mechanisms in Redox Flow Batteries. Nature 2020 579:7798 2020, 579 (7798), 224–228, DOI 10.1038/s41586-020-2081-7.

(10) Wu, B.; Aspers, R. L. E. G.; Kentgens, A. P. M.; Zhao, E. W. Operando Benchtop NMR Reveals Reaction Intermediates and Crossover in Redox Flow Batteries. Journal of Magnetic Resonance 2023, 351, 107448, DOI 10.1016/J.JMR.2023.107448.

(11) Zuo, P.; Li, Y.; Wang, A.; Tan, R.; Liu, Y.; Liang, X.; Sheng, F.; Tang, G.; Ge, L.; Wu, L.; Song, Q.; McKeown, N. B.; Yang, Z.; Xu, T. Sulfonated Microporous Polymer Membranes with Fast and Selective Ion Transport for Electrochemical Energy Conversion and Storage. Angewandte Chemie International Edition 2020, 59 (24), 9564–9573, DOI 10.1002/anie.202000012.
